# Exploring the diversity of galls on *Artemisia indica* induced by *Rhopalomyia* species through morphological and transcriptome analyses

**DOI:** 10.1002/pld3.619

**Published:** 2024-07-02

**Authors:** Seiji Takeda, Makiko Yoza, Sawako Ueda, Sakura Takeuchi, Akiteru Maeno, Tomoaki Sakamoto, Seisuke Kimura

**Affiliations:** ^1^ Graduate School of Life and Environmental Sciences Kyoto Prefectural University Kyoto Japan; ^2^ Biotechnology Research Department, Kyoto Prefectural Agriculture Forestry and Fisheries Technology Center Seika Japan; ^3^ Cell Architecture Laboratory National Institute of Genetics Shizuoka Japan; ^4^ Center for Plant Sciences Kyoto Sangyo University Kyoto Japan; ^5^ Department of Industrial Life Sciences, Faculty of Life Sciences Kyoto Sangyo University Kyoto Japan

**Keywords:** *Artemisia indica*, gall, microCT, *Rhopalomyia*, RNA sequencing

## Abstract

Plant galls generated by insects have highly organized structures, providing nutrients and shelter to the insects living within them. Most research on the physiological and molecular mechanisms of gall development has focused on single galls. To understand the diversity of gall development, we examined five galls with different morphologies generated by distinct species of *Rhopalomyia* (gall midge; Diptera: Cecidomyiidae) on a single host plant of *Artemisia indica* var. *maximowiczii* (Asteraceae). Vasculature developed de novo within the galls, indicating active transport of nutrients between galls and the host plant. Each gall had a different pattern of vasculature and lignification, probably due to differences in the site of gall generation and the gall midge species. Transcriptome analysis indicated that photosynthetic and cell wall–related genes were down‐regulated in leaf and stem galls, respectively, compared with control leaf and stem tissues, whereas genes involved in floral organ development were up‐regulated in all types of galls, indicating that transformation from source to sink organs occurs during gall development. Our results help to understand the diversity of galls on a single herbaceous host plant.

## SIGNIFICANCE STATEMENT

Gall insects manipulate plant organs to generate galls providing nutrients and protection to them. We examined a host herbaceous plant, *Artemisia indica*, where gall midges (*Rhopalomyia* species) generate different types of galls, and by morphological and transcriptome analyses, we found that galls had different characters in lignification and vascular pattern and that transcriptional profiles were dramatically changed from the original source organs.

## 1 INTRODUCTION

Galls are abnormal plant tissues generated by viruses, bacteria, protozoa, oomycetes, fungi, parasitic plants, or animals such as rotifers, nematodes, mites, and insects (Harris & Pitzschke, 2020). These modified plant structures provide nutrients and protection to the organisms that induce the gall (Giron et al., 2016; Price et al., 1987; Stone & Schönrogge, 2003). Many galls generated by insects, such as gall wasps and midges, develop into highly organized structures, indicating that gall‐inducing insects hijack plant development to generate unique structures convenient for them. There are an estimated 21,100 to 211,000 species of gall‐inducing insects with a wide variety of host land plants (Espírito‐Santo & Fernandes, 2007).

Host plants generate galls in response to physical stimuli such as oviposition or feeding and chemical signals such as secreted short peptides, proteins, and phytohormones (Hirano et al., 2024; Korgaonkar et al., 2021; Mapes & Davies, 2001; Straka et al., 2010; Tooker & De Moraes, 2011; Yamaguchi et al., 2012). Transcriptome and metabolome analyses of developing or mature galls have identified genes and molecules potentially playing roles in the maintenance of the gall structure (Bailey et al., 2015; Chen et al., 2018; Markel et al., 2024; Schultz et al., 2019). Many insect‐induced galls resemble ectopic fruits, and some accumulate high concentrations of nutrients including amino acids (Koyama et al., 2004; Suzuki et al., 2009). Transcriptome analyses of galls of *Rhus javanica* and wild grapevine (*Vitis riparia*) showed that genes involved in floral organ development are up‐regulated in these galls compared with control tissues (Hirano et al., 2020; Schultz et al., 2019). For example, the expression of floral homeotic genes in galls indicates that they are involved in transforming vegetative organs, such as leaves, into organs with reproductive features (Krizek & Flether, 2005; Theißen & Saedler, 2001). However, as galls are not identical to the fruits borne by host plants, gall‐inducing insects most likely stimulate and integrate several plant systems such as stress responses and development to create novel structures not typically generated in plants without stimuli from gall‐inducing insects.

Most previous research has been based on single galls induced by one insect species specific to an individual plant species, making these results specific and limited to each type of gall. We previously showed that 38 genes were commonly up‐regulated in four different galls generated on *Artemisia montana*, *Eurya japonica*, *Rhus javanica*, and *Glochidion obovatum*, compared with their expression in control leaves (Takeda et al., 2019). These common genes were involved in the regulation of cell cycle and cytokinesis, lignification, phytohormone signaling, stress responses, and metabolic processes, indicating that these processes are critical for gall development. However, the number of common genes was small due to phylogenetic differences in insect and host plant species. Moreover, many galls are generated on woody plants, which are difficult to handle within the laboratory, making it challenging to explore the molecular mechanisms of gall development. Therefore, establishing a model system for gall development on herbaceous plants is necessary for rearing plants and insects and for genetic analysis through breeding or transgenic techniques.

To understand the diversity of gall development in herbaceous plants, we focused on galls generated on *Artemisia indica* var. *maximowiczii* (Asteraceae) by *Rhopalomyia* species (commonly called gall midges; Diptera: Cecidomyiidae) and examined their morphology and comparative gene expression by RNA sequencing (RNA‐seq). Different *Rhopalomyia* midges generate various types of galls on *A. indica* (Table 1; Ganaha et al., 2007, 2007; Nohara et al., 2007; Sato et al., 2009; Tanaka et al., 2013). Our results highlight the commonality and diversity of gall development in a single host plant, contributing to our understanding of gall development in herbaceous plants to establish a model system for gall development.

**TABLE 1:** Galls used in this study.

Gall name (in Japanese)	Abbreviation	Host organs	Galling midge (Yukawa & Masuda, 1996)
Yomogi‐ha‐eboshi‐fushi	Eboshi	Leaf	*Rhopalomyia yomogicola* Matsumura
Yomogi‐ha‐shiro‐ketama‐fushi	Ketama	Leaf	*Rhopalomyia cinerarius* Monzen
Yomogi‐kuki‐cobu‐fushi	Cobu	Stem	*Rhopalomyia struma* Monzen (*Rhopalomyia yomogi* Shinji)
Yomogi‐kuki‐wata‐fushi	Wata	Stem	*Rhopalomyia giraldii* Kieffer & Trotter (*Rhopalomyia neoartemisiae* Shinji; *Rhopalomyia gossypii* Monzen)
Yomogi‐metsubo‐fushi	Metsubo	Axillary bud	*Rhopalomyia shinjii* Gagne (*Misospatha artemisiae* Shionji; *Misosphatha yomogi* Shinji; *Panteliola ampurila* Monzen)

## 2 RESULTS

**FIGURE 1:**
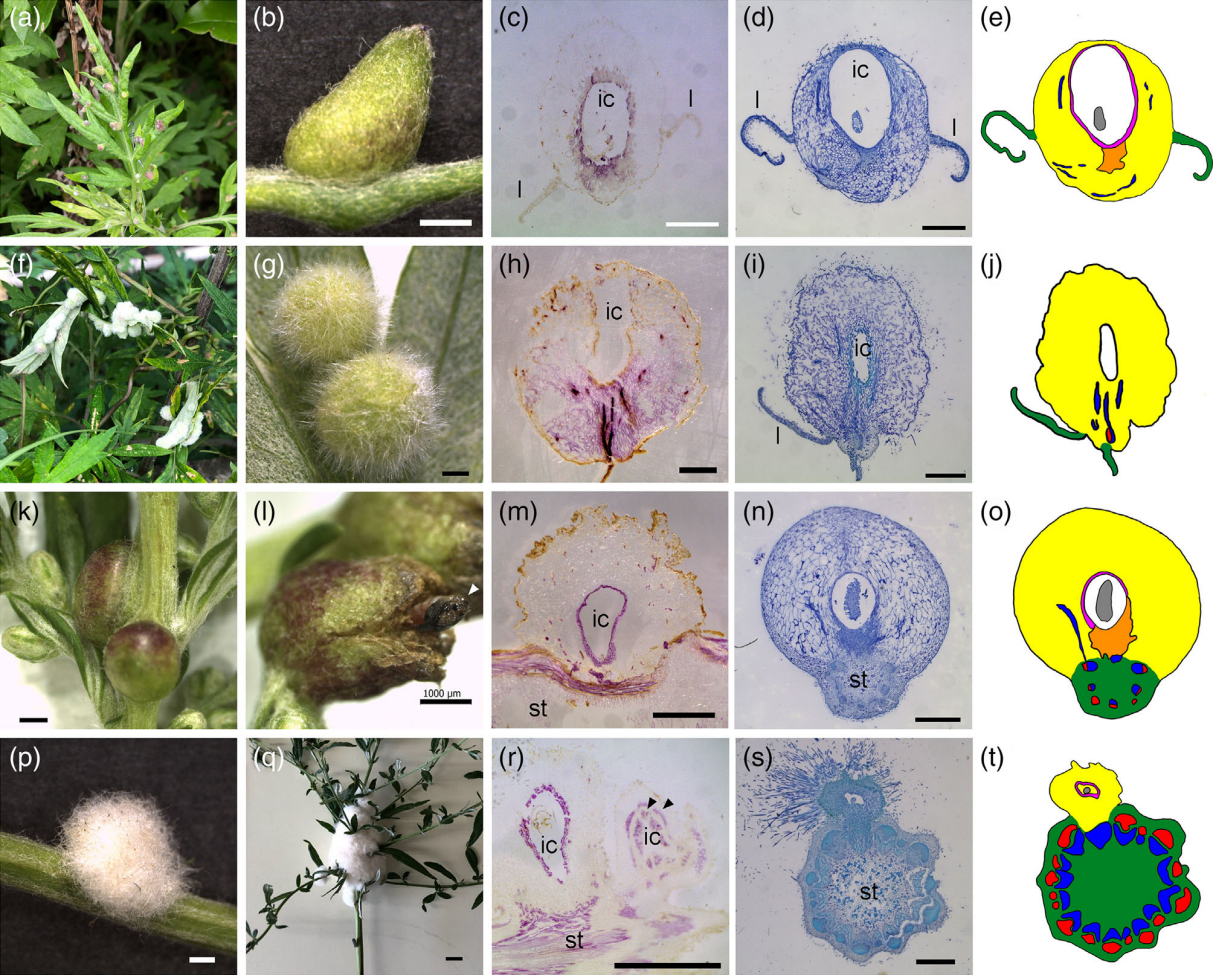
**Morphology of galls on leaves or stems.** (a–e) Yomogi‐ha‐eboshi‐fushi (Eboshi). (f–j) Yomogi‐ha‐shiro‐ketama‐fushi (Ketama). (k–o) Yomogi‐kuki‐cobu‐fushi (Cobu). Arrowhead in (l) indicates an eclosed midge emerging from the gall. (p–t) Yomogi‐kuki‐wata‐fushi (Wata). (c, h, m, r) Longitudinal sections stained with phloroglucinol, showing lignin staining with purple color. Note that two layers of lignification surround the insect chamber in the Wata gall (arrowheads in r). (d, i, n, s) Longitudinal sections stained with toluidine blue. (e, j, o, t) Schematic illustrations of galls. Yellow, gall tissue; green, leaf or stem of the host plant; pink, lignified tissue; red, phloem; blue, xylem; orange, small cells; l, leaf; st, stem; ic, insect chamber. Bars: q, 1 cm; b–d, g–i, k–p, r, s, 1 mm.

We focused on distinct types of galls generated by gall midges (*Rhopalomyia* species) on *A. indica* Willd. var. *maximowiczii* (Nakai) H. Hara (syn. *A. princeps* Pamp.), generally called Japanese mugwort in English and yomogi in Japanese (Table 1). These galls are found in nature at many locations in Japan from spring to autumn. First, we compared four types of galls: two on leaves and two on stems (Figure 1, Table 1). In Japanese, these galls are named by the following convention: host plant name, organ where galls are generated, shape, and “fushi,” meaning a gall. Because they do not have proper names in English, we begin by explaining the morphology of each type of gall using its Japanese name and abbreviation.

### 2.1 Galls on leaves

The first type of gall isolated from *A. indica* leaves was called “yomogi‐ha‐eboshi‐fushi” (hereafter “Eboshi”), meaning a gall with the shape of the traditional Japanese noble headgear (Figure 1a,b; Tanaka et al., 2013). This type of gall penetrated both sides of the leaf (Figure 1a–e) and had a green or purple color on its surface. The galls had a polarity, namely, the upper part was sharp and the lower part was round, creating a strawberry‐like shape (Figure 1b). There was a chamber within the gall housing an insect larva or pupa. The cells surrounding the insect chamber were lignified (Figure 1c). Small cells that lay between the insect chamber and the host leaf tissue likely provide a food source for larvae (Figure 1d,e; Tanaka et al., 2013).

The second type of gall on leaves was named “yomogi‐ha‐shiro‐ketama‐fushi” (Ketama), meaning a round gall with white hairs. As the name indicates, these were covered with white hairs (Figure 1f,g). There was an insect chamber within the gall, but the surrounding cells were less lignified than those of other galls (Figure 1h). The strong, fiber‐like staining of lignin was detected between cells of the host plant and the insect chamber (Figure 1h), showing that vascular bundles with secondary cell walls connect the insect chamber and the host tissue. The parenchyma tissue of the galls was highly vacuolated (Figure 1i).

**FIGURE 2:**
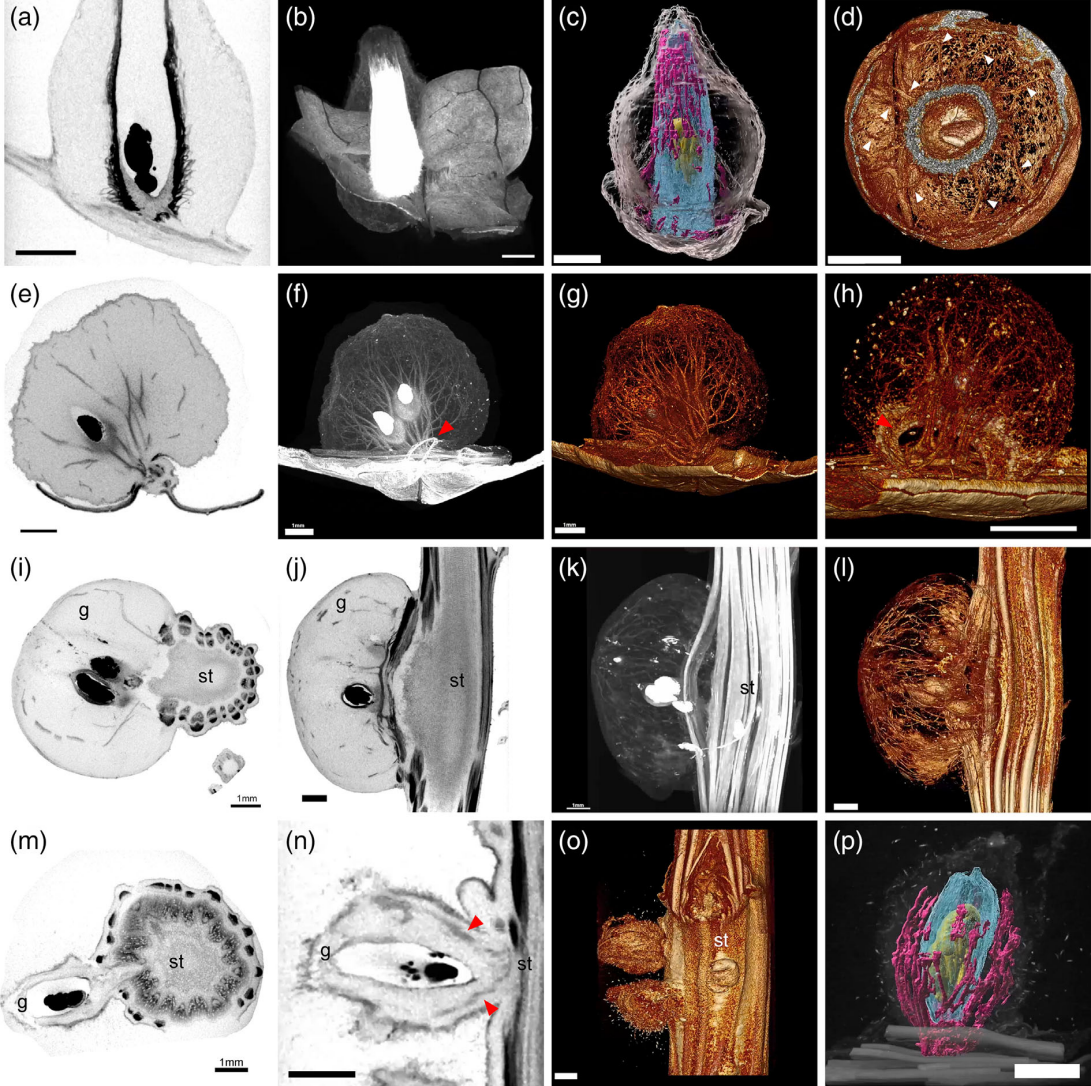
**X‐ray microCT images of galls.** (a–d) Eboshi, (e–h) Ketama, (i–l) Cobu, and (m–p) Wata. (a, e, i, j, m, n) Virtual sections showing inside of the galls. Hard tissues are shown in black. (b, f, k) Maximum intensity projection (3D‐MIP) images of galls with grayscale. Hard tissue is shown in white. (c, p) Pseudo‐colored 3D surface model (3D‐SM) images of galls. Purple, lignified vascular tissue; blue, inset chamber; yellow, insects. (d, g, h, l, o) Three‐dimensional volume rendering (3D‐VR) images of galls with pseudo‐color. (a) Longitudinal section of Eboshi gall showing insects and lignified cells in black. Note that the nutritive tissue below the insect chamber is connected to the host vascular bundles. (b, c) lignified hard cells surround the insect chamber. (d) Transverse image of Eboshi gall showing vascular bundles connecting the outer wall and the insect chamber (arrowheads). (e) Longitudinal section of Ketama gall showing insect and several vascular bundles running in the gall tissue. (f, g) Vasculature running throughout the gall reminiscent of tree branching. (h) De novo vasculature emerges from the leaf vasculature of the host plant and runs through the round hole at the bottom of the gall (red arrowheads in f and h). (i, j) transverse (i) and longitudinal (j) sections of a Cobu gall on a stem showing insects and vascular bundles in the gall tissue. (k, l) Vascular bundles connect the gall tissue and host vasculature of the stem. (m, n) Transverse (m) and longitudinal (n) sections of a Wata gall. (o) Two Wata galls on stem. Note that Wata galls are surrounded by many hairs, but the main galls are much smaller than Cobu galls (compare Figure 2o with Figure 2k). Red arrowheads in (n) show the lignified layer surrounding the outside of the insect chamber, as shown in Figure 1r. (p) Hard lignified vascular bundles running around the insect chamber. Images are representative of multiple galls of the same type. g, gall; st, stem. Bars, 1 mm.

**FIGURE 3:**
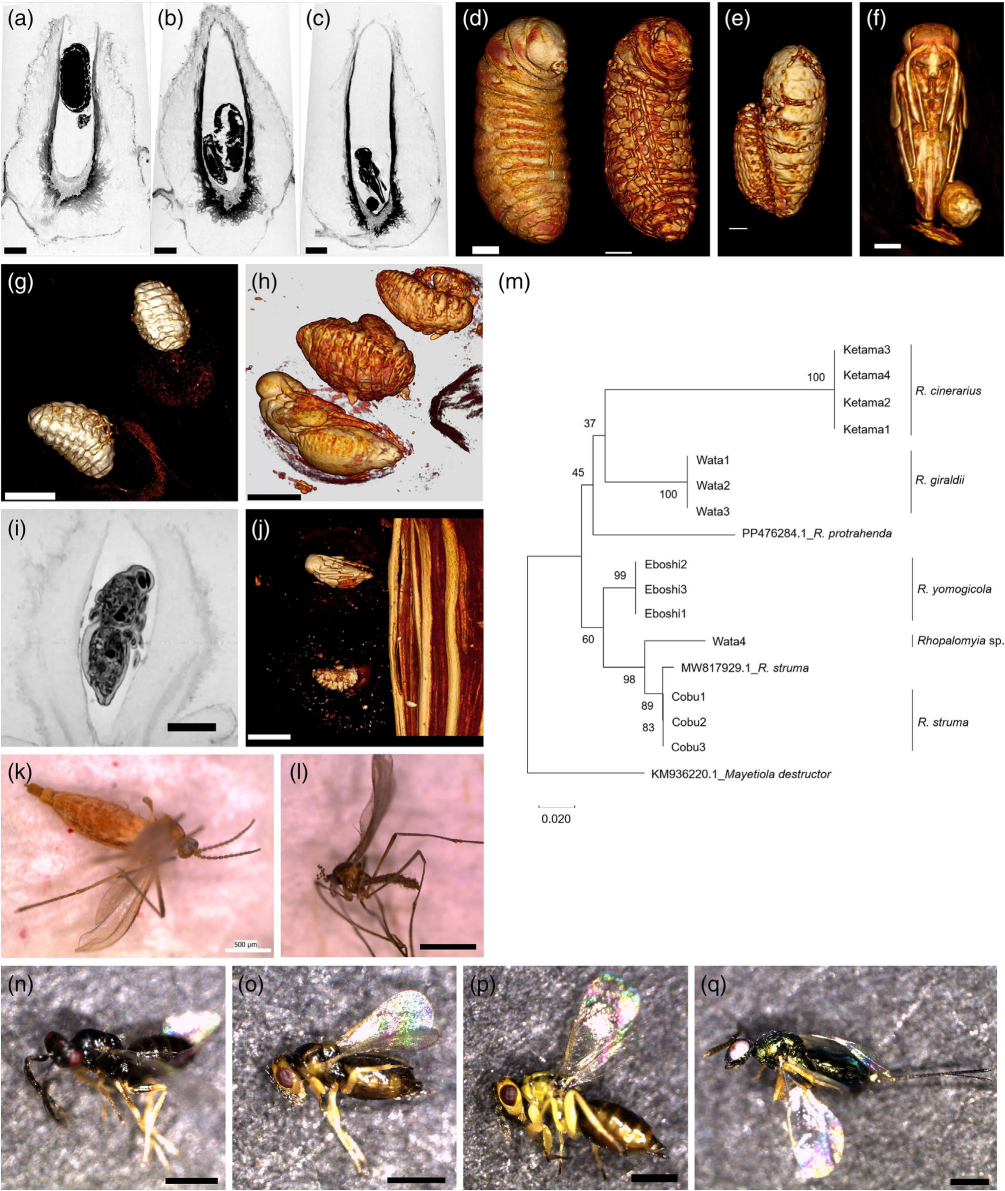
**Larva, pupa, and adult of gall midges and parasitic wasps.** (a–f) Larvae or pupae in Eboshi galls. (g) Larvae in Ketama galls. (h) Pupa and larvae in Cobu galls. Note that at least three insects were found in a single Cobu gall in this case. (i, j) Pupa and larvae in Wata galls. (k) Female adult midge from an Eboshi gall. (l) Male adult midge from a Ketama gall. (m) Phylogenetic tree of midge species emerging from each gall. Note that they are all *Rhopalomyia* species, and three of the four species were classified into monophyletic groups, although one Wata midge was classified into the Cobu clade. For comparison, sequences of the mitochondrial *cytochrome oxidase subunit I* (*COI*) gene from *R. struma* (MW817929.1), *R. protrahenda* (accession number: PP476284.1), and *Myetiola destructor* (KM936220.1) are shown. Bootstrap values are indicated for nodes (1000 replicates). (n) Parasitic wasp that emerged from a Ketama gall (*Aprostocetus* sp., male). (o) Parasitic wasp from a Cobu gall (*Aprostocetus* sp., female). (p, q) parasitic wasps that emerged from Wata galls (p: *Aprostecetus* sp., female; q, *Torymus* sp., female). Bars: a–c, g–k, n–q, .5 mm; d–f, .2 mm; l, 1 mm.

In summary, the two types of galls on leaves possessed different structures in regard to surface hairs, shape, and lignification pattern within the gall (Figure 1e,j). In both gall types, a single gall contained one or two larvae or pupae in a chamber, with the second insect possibly being a parasitic wasp (see Figures 2 and 3).

### 2.2 Galls on stems

The first type of gall on stems was called “yomogi‐kuki‐cobu‐fushi” (Cobu), meaning a round gall generated on a stem. These had few hairs and a hard surface, which was red or brown in color (Figure 1k). We observed that the eclosed midge emerged from the cracked edges of the galls (Figure 1l). Histochemical staining of sections revealed that the cells surrounding the inside of the insect chamber were lignified (Figure 1m), and small cells located below the insect chamber seemed to be a nutrient source for the larva (Figure 1n,o: Tanaka et al., 2013). The number of chambers in the single gall was ranged from one to between one and four chambers in a single gall (Figure 1n; see also Figure 2i,k,l, and Movies 6 and 7).

The second type of gall on stems was named “yomogi‐kuki‐wata‐fushi” (Wata), meaning a cotton gall on a stem (Figure 1p,q). As the name indicates, these galls were covered with long, white hairs. Some galls were small with a round shape (Figure 1p), and the others were as large as 5 cm in diameter (Figure 1q), probably resulting from multiple galls in close proximity (see Figure 2). The cells surrounding the insect chamber were lignified, and two layers of lignified cells surrounded the chamber in some galls (Figure 1r).

Histochemical staining of sections showed that both types of stem gall were initiated from the procambium layer of the stem of the host plant (Figure 1n,o,s,t), indicating that the gall midges used the meristematic cambium cells of the host plants to initiate galls, rather than inducing de‐differentiation of the host plant cells. By contrast, the insect chambers were at a distance from the host vasculature, and de novo vasculature connects the chamber and host vasculature (Figure 1c–e, h–j; see also Figure 2), indicating that initiation of galls varies between host organs where the galls are generated.

### 2.3 MicroCT imaging revealed the internal structure of the galls

To understand the inner structure of the galls, we used non‐destructive X‐ray micro‐computed tomography (microCT). This technique enabled us to extract the hard and soft tissues within the organ using differences in X‐ray transparency and to construct 3D visualizations of both animal and plant specimens (Metscher, 2009; Staedler et al., 2013). The longitudinal virtual section and 3D volume rendering (3D‐VR) and maximum intensity projection images of Eboshi galls showed that the insect chamber was surrounded by hard cells, with a soft tissue below the chamber connecting to the vasculature of the host leaf (Figure 2a,b, Movie 1). Pseudo‐colored 3D surface model images revealed vasculature running around the insect chamber (Figure 2c, Movie 2), whereas transverse 3D‐VR images showed thin vasculature connections between the insect chamber and the inner side of the gall wall (Figure 2d, Movie 3). Compared with Eboshi galls, Ketama galls showed a highly developed vascular pattern running throughout the gall, reminiscent of tree branching (Figure 2e–g, Movie 4). Ketama galls had a ring‐like structure surrounded by hard tissue at the bottom of the gall; vasculature emerged from the host vasculature and ran within the ring, connecting to the insect chamber (Figure 2f,h, Movie 5).

Galls on stems initiated from a part of the vasculature of the host stem (Figure 2i,j,m,n). In Cobu galls, the vasculature of the host stem bended toward the gall, and the insect chamber was located close to the stem (Figure 2i,j, Movie 6). Vascular bundles ran within the gall reminiscent of tree branching, similar to Ketama galls (Figure 2k,l, Movie 7). Both Cobu and Wata galls appeared to initiate from the procambium layer between vascular bundles of the host stem (Figures 2i,m and 1n,s, Movies 6–9). The location of the insect chamber was close to the host stem in both Wata and Cobu galls (Figure 2j,k,n,o). As shown in Figure 1r, there was an outer layer of lignified cells in Wata galls, assumed to be the vascular bundles (Figure 2n, Movie 8). The vascular pattern was simpler in Wata galls than in Cobu galls, running unidirectionally around the insect chamber (Figure 2p, Movie 10).

Together, these results indicate that each type of gall possesses a distinct structure, with differences in lignification, vascular patterning, and the location of the insect chamber, even when galls are generated on the same host organs (leaves or stems).

### 2.4 Morphology and phylogeny of gall midges

These four types of galls are generated by *Rhopalomyia* species (Table 1). MicroCT analysis allowed us to examine the morphology of the larva and pupa inside the gall. Some Eboshi galls carried a single larva or pupa, but others had two insects inside the chamber (Figure 3a–c, Movie 11). The shape and size of the galls with or without parasites were not different (Figure 3a,b), indicating that parasites had no or less effect on gall development; 3D‐VR images showed the morphology of the larvae (Figure 3d–f). Larvae in the insect chamber had particles and a network structure under the skin (Figure 3d, Movie 12). In the gall with two larvae, the larvae were of different sizes (Figure 3e, Movie 13), indicating that they were a midge and a parasitic wasp. Larvae turned into pupae inside the chamber, and in several cases, the pupa appeared to be a parasitic wasp (Figure 3f).

It was difficult to distinguish morphological differences between the adult midges that emerged from galls (Figure 3g–l, Movies 9, 14, and 15). Gall midges of *A. indica* were described previously based on their galls (Table 1; Yukawa & Masuda, 1996), and some were classified based on the sequence of mitochondrial *cytochrome oxidase subunit I* (*COI*, Ganaha et al., 2007, 2007; Nohara et al., 2007; Sato et al., 2009; Skuhravá et al., 2016). Therefore, we investigated the molecular phylogeny of the midges emerging from each gall by comparing the *COI* sequences. A homology search using BLAST with the *COI* sequences as a query indicated that the midges were *Rhopalomyia* species, which were classified into distinct species (Figure 3m). A midge from a Wata gall (Wata 4) was classified in a different cluster from the other Wata midges, close to the Cobu midges. This raises several hypotheses: (1) a small Cobu gall was generated near the Wata gall of this sample, (2) it was an inquiline, or (3) the midge was undergoing speciation. More galls and midges need to be analyzed to clarify these hypotheses. Parasitic wasps, most likely *Aprostocetus* sp. or *Torymus* sp., emerged from some galls (Figure 3n–q; Bae & Jung, 2020; Matsuo et al., 2018). This, together with the microCT data, indicates that parasitism often occurs in the galls; thus, the galls are a remarkable target for parasitic wasps.

### 2.5 Transcriptome analysis

**FIGURE 4:**
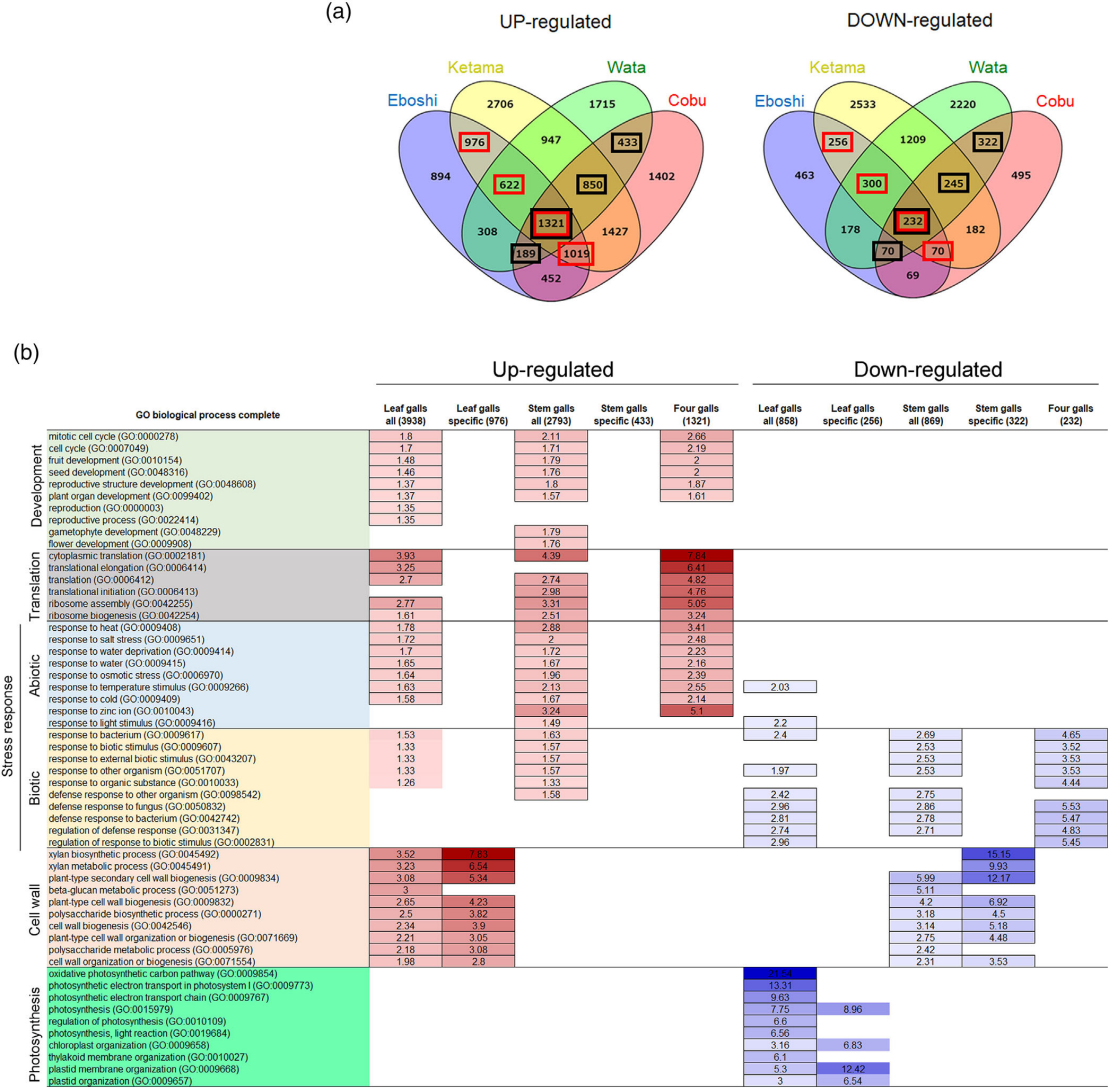
**Transcriptome analysis of four galls on *A. indica* .** (a) Venn diagrams showing the number of up‐ and down‐regulated genes in the four types of galls. Numbers in red and black squares represent genes included in the “all” genes category in leaves and stems, respectively, shown in (b). (b) GO analysis of genes expressed in galls. The first column shows the category and number of genes. Numbers in the table indicate fold enrichment of the GO enrichment analysis, and up‐ and down‐regulated genes are colored in red and blue, respectively. GO terms on the left are colored according to function: development, pale green; translation, gray; abiotic stress response, pale blue; biotic stress response, pale orange; cell wall, pale red; photosynthesis, green.

To understand similarities and differences in the mechanisms of gall development, we compared the genes expressed in the four types of galls. We isolated RNA from galls and control tissues (leaves and stems), performed RNA‐seq, and selected up‐ and down‐regulated genes whose expression level in galls was more than double or less than half that in control tissues, respectively (Figure 4a). Compared with their expression levels in leaf tissue, 3938 and 858 genes were up‐ or down‐regulated in leaf galls, respectively, with 976 and 256 specific to leaf galls. Compared with their expression levels in stem tissue, 2793 and 869 genes were up‐ and down‐regulated in stem galls, respectively, with 433 and 322 genes specific to stem galls. The four types of galls had 1321 and 232 up‐ and down‐regulated genes in common, respectively.

Gene Ontology (GO) analysis showed that genes involved in organ development and cell cycle, translation, and responses to abiotic stress were activated in leaf and stem galls (Figure 4b, Table S1). Transcription factor genes included flowering and development genes, such as the floral homeotic genes *AGAMOUS* and *FRUITFULL*, *ABORTED MICROSPORES* involved in anther development, *FILAMENTOUS FLOWER* associated with adaxial and abaxial patterning, and *PERIANTHIA* associated with floral organ number (Table 2: Yanofsky et al., 1990; Gu et al., 1998; Sorensen et al., 2003; Sawa et al., 1999; Chuang et al., 1999). In leaf galls, photosynthetic genes were down‐regulated (Figure 4b, Table S2), supporting that gall insects modify the original function of the host plant organs and transform them from source to sink organs during leaf gall development. Interestingly, cell wall–related genes were up‐regulated in leaf galls and down‐regulated in stem galls. In both types of gall, genes associated with biotic stress responses were up‐ and down‐regulated, supporting that the defense system of the host plant was disturbed. In the hairy galls (Ketama and Wata), genes involved in cuticle development and metabolic processes were up‐regulated, whereas genes associated with abiotic stress responses were down‐regulated (Tables S3 and S4), supporting again that galls have acquired novel plant organ features.

**TABLE 2:** Transcription factors upregulated in leaf and stem galls.

Gene description	AGI code	Function	Reference
AtMYB69	AT4G33450		
Calmodulin‐binding protein 60 A;CBP60A	AT5G62570		
Zinc‐finger homeodomain protein 6;ZHD6	AT2G18350		
Transcription factor bHLH93, NO FLOWERING IN SD (NFL)	AT5G65640	Flowering	Sharma et al., 2016
ABORTED MICROSPORES;AMS	AT2G16910	Anther development	Sorensen et al., 2003
High mobility group B protein 2;HMGB2	AT1G20693		
AGAMOUS‐LIKE MADS‐box protein AGL53	AT5G27070		
AT‐hook motif nuclear‐localized protein 22;AHL22	AT2G45430	Flowering	Yun et al., 2012
Transcription factor bHLH123;BHLH123	AT3G20640		
Mediator of RNA polymerase II transcription subunit 13;MED1, MACCHI‐BOU2, MAB2	AT1G55325	Auxin response, embryogenesis	Ito et al., 2011
MYB17, LATE MERISTEM IDENTITY2, LMI2	AT3G61250	Floral meristem identity	Pastore et al., 2011
FACT complex subunit SSRP1	AT3G28730		
Ethylene‐responsive transcription factor ERF060	AT4G39780		
EMB1444/LHL1	AT1G06150		
Lysine‐specific demethylase JMJ14	AT4G20400		
NAC domain‐containing protein 7;NAC007	AT1G12260		
Zinc finger protein WIP2	AT3G57670		
Floral homeotic protein AGAMOUS, AG	AT4G18960	Floral organ identity	Yanofsky et al., 1990
Homeobox‐leucine zipper protein ATHB‐40	AT4G36740	Gibberellin homeostasis	Dong et al., 2022
Jumonji (JmjC) domain‐containing protein	AT1G11950		
BEL1‐like homeodomain protein 6;BLH6	AT4G34610	Secondary cell wall development	Liu et al., 2014
bHLH62	AT3G07340		
Auxin response factor 4;ARF4	AT5G60450		
ABA‐INDUCIBLE bHLH‐TYPE;AIB; JAM1	AT2G46510	JA signaling	Nakata et al., 2013
PERIANTHIA;PAN	AT1G68640	Floral organ number	Chuang et al., 1999
Scarecrow‐like transcription factor PAT1	AT5G48150		
bZIP transcription factor 16;bZIP16	AT2G35530	Seedling development	Hsieh et al., 2012
Axial regulator YABBY 1;YAB1;PTN000783201;orthologs FIL	AT2G45190	Adaxial–abaxial patterning	Sawa et al., 1999
Auxin response factor 5;ARF5, MONOPTEROS, MP	AT1G19850	Auxin response, embryogenesis	Hardtke & Berleth, 1998
High mobility group B protein 3;HMGB3;PTN001009907;orthologs	AT1G20696		
SUPPRESSOR OF GAMMA RESPONSE 1;SOG1	AT1G25580	DNA damage response	Waterworth et al., 2022
B‐box type zinc finger family protein;BBX28	AT4G27310		
High mobility group B protein 6;HMGB6;PTN000345582;orthologs	AT4G23800		
Homeobox‐leucine zipper protein ATHB‐13	AT1G69780	Seedling transition	Silva et al., 2016
AGAMOUS‐LIKE MADS‐box protein AGL8, FRUITFULL, FUL	AT5G60910	Fruit development	Gu et al., 1998

### 2.6 Comparison with galls at axillary buds

**FIGURE 5:**
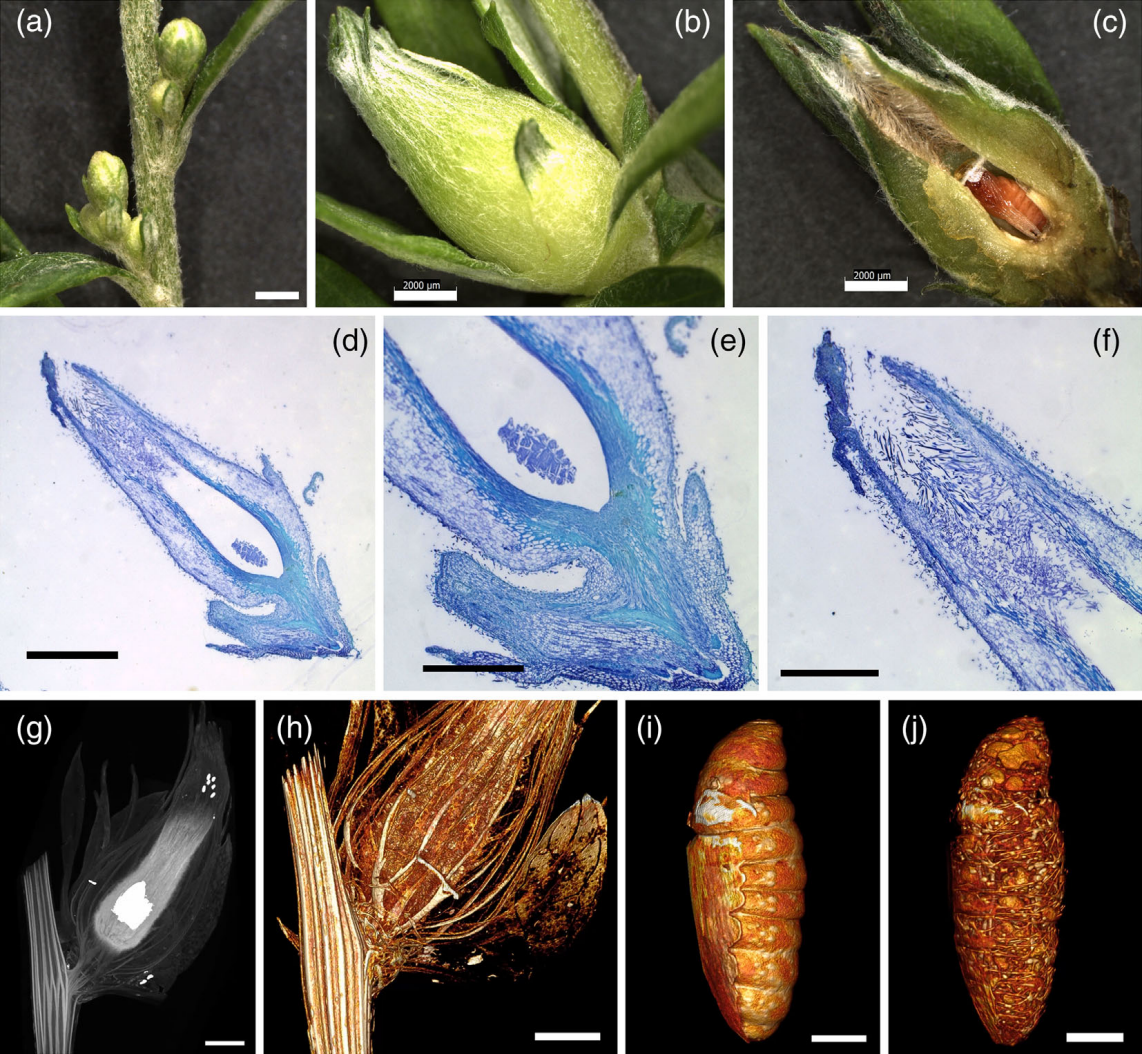
**Morphology of yomogi‐metsubo‐fushi (Metsubo).** (a) Axillary buds of *A. indica* . (b) Metsubo gall generated at the axil. (c) Metsubo gall in (b) cut in half to show a pupa inside. (d–f) Longitudinal section of a Metsubo gall. (e) and (f) are higher magnification images of the bottom and top parts of (d), respectively. Note that Metsubo galls are open at the distal end, which is covered with many hairs (f). (g–j) X‐ray microCT images. (g) Maximum intensity projection (3D‐MIP) image of a Metsubo gall showing the stem vasculature, insect chamber, and insect inside in white. (h) 3D volume rendering (3D‐VR) image showing the development of vasculature from that of the stem. (i, j) Larva inside the insect chamber. (j) Inside the larva body showing a network of fibers and many particles. Bars: a–d, g, h, 2 mm; e, f, 1 mm; i, j, .5 mm.

The fifth type of gall examined in this study was named yomogi‐metsubo‐fushi (Metsubo) and was generated at the stem axil (Table 1). Metsubo galls resembled floral buds but the shape was different, and the size was much larger than floral buds at the axil (Figure 5a,b). The inside surface of the distal end of the gall was covered with many hairs, likely to protect the insects inside from predators or rain (Figure 5c–f). Vascular bundles ran along the proximo‐distal axis in the parenchyma of the gall, connecting to the host vasculature of the stem (Figure 5g,h, Movie 16). There was one larva or pupa inside the gall, and a network structure and particles lay under the skin of the larva body (Figure 5c,i,j).

We examined the genes expressed in Metsubo galls by RNA‐seq analysis; 6700 and 3240 genes were up‐ and down‐regulated, respectively, in galls compared with control axillary buds. By comparing these genes to the genes up‐ or down‐regulated in all four types of leaf and stem galls, we identified 926 and 124 genes that were up‐ or down‐regulated in all five types of gall. GO analysis showed that the up‐regulated genes were involved in the development and abiotic stress responses (Table S5). Down‐regulated genes were involved in responding to biotic stress (Table S6), supporting that gall insects suppress the resistance systems of their host plants to generate galls.

## 3 DISCUSSION

We identified similarities and differences in galls generated on *A. indica* by *Rhopalomyia* species by examining morphology and gene expression. We focused on a single host plant where distinct *Rhopalomyia* species generate different types of galls. Each type of gall appeared to have evolved independently, since our data showed little relationship between the features of galls (e.g., site of gall generation [leaf or stem] and vascular pattern) and midge phylogeny (Figure 3m).

In the two types of galls on leaves, Eboshi and Ketama, the insect chambers were located at a distance from the vasculature of the host plants; de novo vascular bundles connected the insect chamber and the vasculature of the host plant (Figure 1), indicating the transport of water and nutrients. Transport from host plants to gall tissues seems likely, with several studies showing that water‐soluble nutrients can be transported from gall tissues to host plants (Chen et al., 2020: Kutsukake et al., 2012). Therefore, proper development of the vascular system is critical for gall development and function. In the two types of galls on stems, Cobu and Wata, the insect chambers were very close to the host vasculature (Figure 1). Histochemical staining of sections showed that Wata galls are generated from vascular cambium (Figure 1s,t), indicating that galls are initiated from the meristematic cells of the host plant rather than by inducing de‐differentiation of the differentiated cells. Plant cells generally possess high regeneration ability, with differentiation pathways initiated from either undifferentiated or differentiated somatic cells (Ikeuchi et al., 2016). In some galls, including Eboshi galls, larvae accumulate much higher concentrations of auxin and cytokinin than gall tissues (Hirano et al., 2020; Tanaka et al., 2013), indicating that the larvae secrete these phytohormones to induce division and differentiation of host plant cells. We determined that the *MONOPTEROS* gene, encoding a transcription factor mediating embryo axis and vascular development through auxin signaling, is up‐regulated in all four types of galls (Table 2; Hardtke & Berleth, 1998). Together, phytohormones, especially auxin and cytokinin, are key molecules for initiating and maintaining gall development, as suggested in many gall studies.

Genes related to photosynthesis and cell wall biogenesis were down‐regulated in leaf and stem galls, respectively, supporting that galls do not retain the original leaf and stem functions as source and transport organs. By contrast, genes involved in the development of reproductive organs were up‐regulated in all four types of galls, indicating that the transition from vegetative to reproductive organs occurs during gall development, as indicated by previous studies (Hirano et al., 2020; Schultz et al., 2019; Takeda et al., 2019). Genes related to biotic stress responses were both up‐ and down‐regulated in the four types of galls, indicating that gall insects change the status of the plant defense system. How insects regulate the expression and suppression of these genes remains a mystery; if we can clarify this mechanism, we could generate ectopic fruit‐like structures on vegetative tissues for use as a food source or crops with tolerance to insect pests. Plants with galls show greater resistance to cold injury (Rocha et al., 2013) so galls can provide indirect benefits to the host plants.

Gall development can be divided into initiation and maintenance processes. Most research including our work here has focused on the maintenance of galls since it is very difficult to find galls at early developmental stages in the field. To examine the initiation process, the rearing of both host plants and gall insects is required. A micromoth that transforms from a leaf miner to a gall inducer during larvae development, and the weevil oviposition process, have provided insights into gall initiation (Barnewall & De Clearck‐Floate, 2012; Guiguet et al., 2018). The Ab‐GALFA method, which uses extracts from gall aphids and the model plant *Arabidopsis thaliana*, enables us to investigate cellular dynamics and gene function in response to chemical stimuli from gall insects (Hirano et al., 2023); however, *A. thaliana* does not generate galls. We therefore propose *A. indica* as a good herbaceous model for gall development, since it is easy to find in nature and to cultivate in the laboratory, and transgenic methods have been established for related species such as *A. annua* (Hassani et al., 2023). *A. indica* produces functional ingredients such as artemisinin and lutein (Komuro et al., 2017; Mannan et al., 2011), and our preliminary data show that Wata galls possess antioxidant activity similar to *A. indica* leaves, so the gall system of *A. indica* will be valuable for both basic and applied research in the future.

## 4 EXPERIMENTAL PROCEDURES

### 4.1 Gall samples and histochemical sections

Galls of *Artemisia indica* Willd. var. *maximowiczii* (Nakai) H. Hara (syn. *Artemisia princeps* Pamp.) were collected from the university field of the Seika campus of Kyoto Prefectural University (Seika city, Kyoto, Japan) from April to October 2018, 2019, and 2020. For histochemical sections, galls were dissected into 5‐ to 10‐mm squares and fixed in FAA (50% ethanol, 5% acetic acid, 10% formaldehyde, v/v) under vacuum for 15 min, followed by incubation for 4 h at room temperature. The samples were dehydrated through an ethanol series (50%, 60%, 70%, 80%, 90%, and 99.5%, v/v) and an ethanol–lemosol series (ethanol/lemosol = 100/0, 75/25, 50/50, 25/75, and 0/100) and replaced with wax using Paraplast Plus (Sigma‐Aldrich, Japan). These galls were placed in molds and kept at room temperature, and sections with 10‐ to 20‐μm thickness were prepared using a rotary microtome (Yamato Kohki Industrial, Japan) or RM2125 RTS (Leica, Germany). The sections were deparaffinized using lemosol and ethanol and stained with .05% (w/v) toluidine blue or 3% (w/v in HCl‐ethanol) phloroglucinol. Images were captured with an S8AP0 stereomicroscope equipped with an EC3 device or a 2500 DM microscope equipped with a DFC450C camera (Leica, Germany).

### 4.2 MicroCT imaging and histology

Galls were fixed in FAA solution, and after subsequent immersion in an ethanol series, the samples were soaked in contrast agent, 3% (w/v) phosphotungstic acid in 70% (v/v) ethanol solution, or a 1:3 mixture of Lugol's solution and deionized distilled water, as previously described (Degenhardt et al., 2010; Metscher, 2009; Staedler et al., 2013; Tsuda et al., 2017). The samples were then scanned using two X‐ray microCT instruments, ScanXmate‐E090S105 and ScanXmate‐CF110TSH320/460 (Comscantechno Co., Ltd., Kanagawa, Japan). Specimens were scanned at one time (normal scan) or were divided into two parts for higher resolution (multi‐step) scans. Scanned data were reconstructed to tiff format files by coneCTexpress (Comscantechno). Pretreatment and scan conditions are shown in Table S5. Three‐dimensional tomographic images were obtained using OsiriX MD software (Pixmeo, Switzerland). Three‐dimensional surface models were created using Imaris v10.0 (Bitplane, Switzerland). Finally, each video was edited using Premiere Pro (Adobe).

### 4.3 Phylogeny of *Rhopalomyia* species

Galls attached to host plants were cut and grown in a plant box under 16‐h light and 8‐h dark at 25°C, and eclosed midges were immersed in 99.5% (v/v) ethanol. DNA was extracted using a DNeasy Blood and Tissue kit (QIAGEN, Netherlands), and a barcoding region of the mitochondrial *cytochrome oxidase subunit I* (*COI*) gene was amplified by PCR using ExTaq (TaKaRa Bio, Japan) and newly designed primers (Eulo_F1: 5′‐DKTCAACMAATCATAAAKATATTGG‐3′ and Eulo_R2: 5′‐TADACYTCNGGRTGNCCRAAAAAYCA‐3′), which amplify the same region and length (658 bp) as the primer set LCO1490 + HCO2198 (Folmer et al., 1994), under the following conditions: 94°C for 3 min, 40 cycles of 94°C for 30 s, 55°C for 30 s, and 72°C for 45 s, followed by 72°C for 5 min. Amplified fragments were purified using a Wizard SV Gel and PCR clean‐up system (Promega, USA), and sequencing was performed by Macrogen Japan (https://macrogen-japan.co.jp/). Mesquite and MEGA X were used for alignment and phylogenetic analysis, respectively (Kumar et al., 2018; Maddison & Maddison, 2023). The statistical method was maximum likelihood, evolutionary distances were calculated using the Tamura 3‐parameter model (Tamura, 1992), and resulting trees were subjected to bootstrap analysis with 1000 replications.

### 4.4 Transcriptome analysis

Three independent samples were used for RNA extraction and RNA seq. Galls and control samples were collected in the field on April 24, 2018, for Eboshi and leaves; July 29, 2019, for Ketama, Wata, leaves, and stems; July 22, 2020, for Metsubo and axillary buds; and August 14, 2020, for Cobu and stem, immediately frozen in the liquid nitrogen, and kept at −80°C until RNA extraction. Approximately .05 g of tissue was used for RNA extraction according to a modified protocol with an RNeasy Plant Mini Kit (QIAGEN, Germany; Brunner et al., 2004). After the RNA integrity was confirmed by running samples on an Agilent RNA 6000 Nano Chip (Agilent Technologies, USA), .5 μg of each total RNA sample was used for library preparation for RNA‐seq analysis. Libraries were prepared using an Illumina TruSeq Stranded mRNA LT Sample Kit according to the manufacturer's instructions (Illumina, USA). The pooled libraries were subsequently sequenced on an Illumina NextSeq500 sequencing platform, and single‐end reads that were 76 bp long were obtained. These reads were assembled into transcriptome contigs using Trinity with default settings (Li & Durbin, 2009), resulting in 550,118 transcript contigs with median contig length and average contig length of 381 and 616.26 nt, respectively. BLASTX searches of the contigs against non‐redundant protein sequences from the NCBI RefSeq (nr) database were conducted using DIAMOND software (Buchfink et al., 2015) to find similar protein sequences. The reads were mapped to the de novo assembled RNA contigs using Burrows‐Wheeler Aligner (https://github.com/lh3/bwa), and the count data were then subjected to a trimmed mean of M‐value normalization in EdgeR (Robinson et al., 2010). Transcript expression and digital gene expression were defined using the edgeR GLM approach.

RNA sequences have been deposited in the DDBJ database (see data availability section). Eboshi data from *A. montana* were deposited during previous work (accession number DRA008530; Takeda et al., 2019). Up‐ and down‐regulated genes were selected by summing those with a false discovery rate < .01, a sum (total number of mapped reads) > 1, and log_2_FC > 1 (up‐regulated) or log_2_FC < −1 (down‐regulated). The PANTHER (10.5281/zenodo.10536401 Released 2024‐01‐17) classification system through the TAIR (https://www.arabidopsis.org/) database (Mi et al., 2019, 2019; The Gene Ontology Consortium, 2017; Thomas et al., 2022) was used for GO analysis. Venn diagrams were drawn using Venny 2.0.2 (Oliveros, 2007–2015).

## CONFLICT OF INTEREST STATEMENT

The authors declare no competing or financial interests.

## Supporting information


**Table S1.** GO analysis of up‐regulated genes in four types of galls.
**Table S2.** GO analysis of down‐regulated genes in four types of galls.
**Table S3.** GO analysis up‐regulated in hairy galls (Ketama and Wata).
**Table S4.** GO analysis down‐regulated in hairy galls (Ketama and Wata).
**Table S5.** GO analysis of up‐regulated genes in five types of galls.
**Table S6.** GO analysis of down‐regulated genes in five types of galls.
**Table S7.** Sample preparation and scanning protocol for each specimen.

**Movies S1.** X‐ray microCT movies.

**Movies S2.** Eboshi.

**Movies S3.** Eboshi.

**Movies S4.** Ketama.

**Movies S5.** Ketama.

**Movies S6.** Cobu.

**Movies S7.** Cobu.

**Movies S8.** Wata.

**Movies S9.** Wata.

**Movies S10.** Wata.

**Movies S11.** Eboshi insect.

**Movies S12.** Eboshi insect.

**Movies S13.** Eboshi insect.

**Movie S14.** Ketama insect.

**Movie S15.** Cobu insect.

**Movie S16.** Metsubo.

## Data Availability

RNA sequences have been deposited in the DDBJ database under accession numbers DRA017567 (Wata), DRA017568 (Ketama), DRA017569 (Cobu), and DRA017570 (Metsubo), and the sequences of the barcoding region of the mitochondrial *COI* gene under the numbers LC810452 to LC810465.
