# Supplementary material for: Exploring the diversity of galls on Artemisia indica induced by Rhopalomyia species through morphological and transcriptome analyses
Source: Plant Direct. 2024 Jul 2;8(7):e619. doi: 10.1002/pld3.619 (PMC11219473; doi:10.1002/pld3.619)
Supplement: Supplementary file 1 — Table S1. GO analysis of up‐regulated genes in four types of galls. Table S2. GO analysis of down‐regulated genes in four types of galls. Table S3. GO analysis up‐regulated in hairy galls (Ketama and Wata). Table S4. GO analysis down‐regulated in hairy galls (Ketama and Wata). Table S5. GO analysis of up‐regulated genes in five types of galls. Table S6. GO analysis of down‐regulated genes in five types of galls. Table S7. Sample preparation and scanning protocol for each specimen. [file PLD3-8-e619-s008.docx]

**Table S1. GO analysis of up-regulated genes in four galls**

| GO biological process | Fold Enrichment | P-value |
| --- | --- | --- |
| gluconeogenesis (GO:0006094) | 10.86 | 7.62E-05 |
| maturation of LSU-rRNA from tricistronic rRNA transcript (SSU-rRNA, 5.8S rRNA, LSU-rRNA) (GO:0000463) | 9.86 | 8.10E-03 |
| hexose biosynthetic process (GO:0019319) | 9.61 | 2.18E-04 |
| protein refolding (GO:0042026) | 8.67 | 1.53E-05 |
| ATP biosynthetic process (GO:0006754) | 8.2 | 2.74E-04 |
| proton motive force-driven ATP synthesis (GO:0015986) | 8.2 | 2.74E-04 |
| glucose metabolic process (GO:0006006) | 7.69 | 6.40E-06 |
| ATP metabolic process (GO:0046034) | 7.67 | 1.73E-04 |
| cytoplasmic translation (GO:0002181) | 7.67 | 4.57E-11 |
| purine ribonucleoside triphosphate biosynthetic process (GO:0009206) | 7.62 | 6.29E-05 |
| ribonucleoside triphosphate biosynthetic process (GO:0009201) | 7.61 | 8.54E-07 |
| ribosomal large subunit assembly (GO:0000027) | 7.49 | 4.80E-02 |
| purine nucleoside triphosphate biosynthetic process (GO:0009145) | 7.43 | 8.18E-05 |
| ribonucleoside triphosphate metabolic process (GO:0009199) | 7.3 | 5.25E-07 |
| purine ribonucleoside triphosphate metabolic process (GO:0009205) | 7.24 | 3.84E-05 |
| nucleoside triphosphate biosynthetic process (GO:0009142) | 6.94 | 3.03E-06 |
| purine nucleoside triphosphate metabolic process (GO:0009144) | 6.66 | 1.01E-04 |
| monosaccharide biosynthetic process (GO:0046364) | 6.64 | 2.78E-04 |
| long-chain fatty acid biosynthetic process (GO:0042759) | 6.54 | 1.63E-02 |
| translational elongation (GO:0006414) | 6.41 | 7.34E-03 |
| glycolytic process (GO:0006096) | 6.41 | 1.59E-04 |
| nucleoside triphosphate metabolic process (GO:0009141) | 6.21 | 5.46E-06 |
| maturation of LSU-rRNA (GO:0000470) | 6.09 | 1.13E-02 |
| chaperone cofactor-dependent protein refolding (GO:0051085) | 6.07 | 1.85E-03 |
| 'de novo' post-translational protein folding (GO:0051084) | 6.07 | 1.85E-03 |
| oxidative phosphorylation (GO:0006119) | 5.95 | 6.20E-05 |
| water transport (GO:0006833) | 5.87 | 3.85E-02 |
| fluid transport (GO:0042044) | 5.87 | 3.85E-02 |
| protein folding (GO:0006457) | 5.87 | 2.47E-18 |
| ribosomal large subunit biogenesis (GO:0042273) | 5.85 | 1.58E-07 |
| adaxial/abaxial pattern specification (GO:0009955) | 5.76 | 7.64E-03 |
| lipid oxidation (GO:0034440) | 5.71 | 3.41E-03 |
| hexose metabolic process (GO:0019318) | 5.68 | 6.37E-07 |
| chaperone-mediated protein folding (GO:0061077) | 5.59 | 1.38E-04 |
| mitochondrial ATP synthesis coupled electron transport (GO:0042775) | 5.55 | 2.54E-02 |
| aerobic electron transport chain (GO:0019646) | 5.55 | 2.54E-02 |
| 'de novo' protein folding (GO:0006458) | 5.5 | 5.03E-03 |
| ATP synthesis coupled electron transport (GO:0042773) | 5.4 | 6.07E-03 |
| ribosome assembly (GO:0042255) | 5.2 | 1.74E-03 |
| post-transcriptional gene silencing (GO:0016441) | 5.2 | 4.42E-02 |
| pyruvate metabolic process (GO:0006090) | 5.14 | 8.34E-05 |
| respiratory electron transport chain (GO:0022904) | 5.12 | 4.65E-03 |
| ribonucleotide biosynthetic process (GO:0009260) | 5.08 | 8.06E-09 |
| purine ribonucleotide biosynthetic process (GO:0009152) | 5.06 | 9.71E-07 |
| alpha-amino acid catabolic process (GO:1901606) | 5.02 | 5.29E-05 |
| monocarboxylic acid catabolic process (GO:0072329) | 4.96 | 6.56E-03 |
| ribose phosphate biosynthetic process (GO:0046390) | 4.9 | 1.88E-08 |
| long-chain fatty acid metabolic process (GO:0001676) | 4.86 | 1.74E-02 |
| glutamine family amino acid metabolic process (GO:0009064) | 4.86 | 1.74E-02 |
| translation (GO:0006412) | 4.84 | 1.95E-38 |
| peptide biosynthetic process (GO:0043043) | 4.79 | 5.25E-38 |
| translational initiation (GO:0006413) | 4.7 | 2.40E-02 |
| peptide metabolic process (GO:0006518) | 4.66 | 2.51E-38 |
| ribonucleotide metabolic process (GO:0009259) | 4.6 | 1.09E-09 |
| purine nucleotide biosynthetic process (GO:0006164) | 4.59 | 1.40E-06 |
| amide biosynthetic process (GO:0043604) | 4.55 | 5.68E-39 |
| purine ribonucleotide metabolic process (GO:0009150) | 4.53 | 1.08E-07 |
| amide metabolic process (GO:0043603) | 4.46 | 1.50E-44 |
| purine-containing compound biosynthetic process (GO:0072522) | 4.45 | 2.56E-06 |
| monosaccharide metabolic process (GO:0005996) | 4.43 | 5.64E-06 |
| nucleotide biosynthetic process (GO:0009165) | 4.39 | 2.88E-08 |
| amino acid catabolic process (GO:0009063) | 4.38 | 7.88E-04 |
| ribose phosphate metabolic process (GO:0019693) | 4.37 | 4.20E-09 |
| nucleoside phosphate biosynthetic process (GO:1901293) | 4.34 | 3.85E-08 |
| aerobic respiration (GO:0009060) | 4.32 | 8.73E-06 |
| organic acid catabolic process (GO:0016054) | 4.21 | 7.83E-08 |
| carboxylic acid catabolic process (GO:0046395) | 4.21 | 7.83E-08 |
| small molecule catabolic process (GO:0044282) | 4.06 | 1.97E-10 |
| purine nucleotide metabolic process (GO:0006163) | 4.05 | 2.58E-09 |
| energy derivation by oxidation of organic compounds (GO:0015980) | 3.99 | 1.81E-06 |
| cellular respiration (GO:0045333) | 3.97 | 1.24E-05 |
| purine-containing compound metabolic process (GO:0072521) | 3.86 | 2.96E-09 |
| nucleotide metabolic process (GO:0009117) | 3.85 | 1.74E-10 |
| nucleoside phosphate metabolic process (GO:0006753) | 3.67 | 8.42E-10 |
| cellular lipid catabolic process (GO:0044242) | 3.66 | 1.61E-02 |
| nucleobase-containing small molecule metabolic process (GO:0055086) | 3.66 | 1.23E-13 |
| lipid catabolic process (GO:0016042) | 3.5 | 1.69E-02 |
| fatty acid biosynthetic process (GO:0006633) | 3.4 | 1.14E-03 |
| ribosome biogenesis (GO:0042254) | 3.2 | 4.12E-09 |
| protein-RNA complex assembly (GO:0022618) | 3.19 | 3.52E-02 |
| carbohydrate catabolic process (GO:0016052) | 3.09 | 1.61E-04 |
| fatty acid metabolic process (GO:0006631) | 3.02 | 3.44E-06 |
| ribonucleoprotein complex biogenesis (GO:0022613) | 2.88 | 1.91E-08 |
| response to heat (GO:0009408) | 2.85 | 3.13E-07 |
| organophosphate biosynthetic process (GO:0090407) | 2.7 | 5.15E-05 |
| monocarboxylic acid metabolic process (GO:0032787) | 2.65 | 1.84E-11 |
| monocarboxylic acid biosynthetic process (GO:0072330) | 2.64 | 1.30E-02 |
| organophosphate metabolic process (GO:0019637) | 2.61 | 3.36E-08 |
| cytoskeleton organization (GO:0007010) | 2.58 | 1.46E-03 |
| organonitrogen compound biosynthetic process (GO:1901566) | 2.55 | 9.23E-31 |
| rRNA metabolic process (GO:0016072) | 2.52 | 1.70E-02 |
| rRNA processing (GO:0006364) | 2.51 | 3.43E-02 |
| gene expression (GO:0010467) | 2.46 | 1.97E-22 |
| cellular nitrogen compound biosynthetic process (GO:0044271) | 2.31 | 8.67E-24 |
| ncRNA metabolic process (GO:0034660) | 2.3 | 1.08E-04 |
| carbohydrate derivative biosynthetic process (GO:1901137) | 2.25 | 1.26E-04 |
| alpha-amino acid metabolic process (GO:1901605) | 2.24 | 1.08E-02 |
| carboxylic acid metabolic process (GO:0019752) | 2.23 | 8.72E-17 |
| carboxylic acid biosynthetic process (GO:0046394) | 2.19 | 8.75E-05 |
| organic acid biosynthetic process (GO:0016053) | 2.18 | 5.68E-05 |
| organic substance catabolic process (GO:1901575) | 2.13 | 1.69E-18 |
| ncRNA processing (GO:0034470) | 2.13 | 3.34E-02 |
| amino acid metabolic process (GO:0006520) | 2.1 | 8.35E-04 |
| small molecule biosynthetic process (GO:0044283) | 2.09 | 4.36E-07 |
| carbohydrate derivative metabolic process (GO:1901135) | 2.05 | 6.89E-07 |
| nucleobase-containing compound biosynthetic process (GO:0034654) | 2.05 | 1.22E-03 |
| macromolecule biosynthetic process (GO:0009059) | 2.02 | 8.18E-19 |
| response to salt stress (GO:0009651) | 2.02 | 6.12E-04 |
| catabolic process (GO:0009056) | 2.01 | 4.46E-18 |
| modification-dependent protein catabolic process (GO:0019941) | 2.01 | 2.08E-02 |
| proteolysis involved in protein catabolic process (GO:0051603) | 2 | 5.12E-03 |
| modification-dependent macromolecule catabolic process (GO:0043632) | 2 | 1.69E-02 |
| carbohydrate metabolic process (GO:0005975) | 1.97 | 6.41E-06 |
| cellular biosynthetic process (GO:0044249) | 1.95 | 1.03E-25 |
| embryo development ending in seed dormancy (GO:0009793) | 1.95 | 3.10E-03 |
| cellular catabolic process (GO:0044248) | 1.93 | 1.08E-05 |
| organonitrogen compound catabolic process (GO:1901565) | 1.93 | 1.39E-06 |
| cell growth (GO:0016049) | 1.91 | 2.62E-02 |
| embryo development (GO:0009790) | 1.91 | 2.23E-03 |
| oxoacid metabolic process (GO:0043436) | 1.91 | 1.06E-12 |
| organic acid metabolic process (GO:0006082) | 1.9 | 1.94E-13 |
| proteolysis (GO:0006508) | 1.87 | 2.82E-03 |
| protein catabolic process (GO:0030163) | 1.87 | 3.64E-02 |
| macromolecule catabolic process (GO:0009057) | 1.86 | 2.70E-04 |
| cell cycle (GO:0007049) | 1.85 | 2.18E-02 |
| organic substance biosynthetic process (GO:1901576) | 1.84 | 7.77E-30 |
| small molecule metabolic process (GO:0044281) | 1.84 | 2.74E-18 |
| response to temperature stimulus (GO:0009266) | 1.77 | 1.07E-04 |
| biosynthetic process (GO:0009058) | 1.76 | 5.20E-27 |
| fruit development (GO:0010154) | 1.68 | 3.69E-03 |
| response to osmotic stress (GO:0006970) | 1.68 | 3.76E-02 |
| seed development (GO:0048316) | 1.68 | 6.50E-03 |
| organic substance transport (GO:0071702) | 1.63 | 4.04E-02 |
| cellular component biogenesis (GO:0044085) | 1.62 | 9.38E-05 |
| cellular localization (GO:0051641) | 1.61 | 3.54E-02 |
| organonitrogen compound metabolic process (GO:1901564) | 1.56 | 1.17E-21 |
| post-embryonic development (GO:0009791) | 1.55 | 6.33E-05 |
| protein metabolic process (GO:0019538) | 1.52 | 1.80E-11 |
| cellular nitrogen compound metabolic process (GO:0034641) | 1.51 | 2.26E-11 |
| phosphorus metabolic process (GO:0006793) | 1.5 | 1.96E-02 |
| cellular component organization or biogenesis (GO:0071840) | 1.5 | 2.76E-07 |
| reproductive structure development (GO:0048608) | 1.49 | 8.71E-03 |
| reproductive system development (GO:0061458) | 1.49 | 8.86E-03 |
| primary metabolic process (GO:0044238) | 1.46 | 1.54E-30 |
| reproductive process (GO:0022414) | 1.44 | 3.44E-03 |
| developmental process involved in reproduction (GO:0003006) | 1.44 | 2.21E-02 |
| cellular metabolic process (GO:0044237) | 1.44 | 2.61E-23 |
| reproduction (GO:0000003) | 1.44 | 3.85E-03 |
| cellular component organization (GO:0016043) | 1.41 | 7.50E-03 |
| transport (GO:0006810) | 1.41 | 4.42E-02 |
| organic substance metabolic process (GO:0071704) | 1.4 | 1.50E-32 |
| metabolic process (GO:0008152) | 1.38 | 7.58E-34 |
| nitrogen compound metabolic process (GO:0006807) | 1.38 | 9.01E-17 |
| cellular process (GO:0009987) | 1.36 | 3.36E-45 |
| response to stress (GO:0006950) | 1.35 | 2.31E-06 |
| nucleobase-containing compound metabolic process (GO:0006139) | 1.35 | 1.33E-02 |
| response to abiotic stimulus (GO:0009628) | 1.33 | 1.03E-03 |
| multicellular organism development (GO:0007275) | 1.32 | 1.73E-03 |
| multicellular organismal process (GO:0032501) | 1.3 | 4.11E-03 |
| macromolecule metabolic process (GO:0043170) | 1.29 | 3.31E-08 |
| anatomical structure development (GO:0048856) | 1.27 | 3.92E-03 |
| developmental process (GO:0032502) | 1.27 | 2.50E-03 |
| response to stimulus (GO:0050896) | 1.21 | 2.47E-04 |
| Unclassified (UNCLASSIFIED) | 0.64 | 0.00E+00 |
| regulation of biosynthetic process (GO:0009889) | 0.64 | 5.98E-03 |
| regulation of cellular biosynthetic process (GO:0031326) | 0.62 | 2.12E-03 |
| regulation of macromolecule biosynthetic process (GO:0010556) | 0.59 | 1.29E-03 |
| regulation of RNA metabolic process (GO:0051252) | 0.58 | 4.39E-04 |
| regulation of DNA-templated transcription (GO:0006355) | 0.52 | 2.22E-05 |
| regulation of RNA biosynthetic process (GO:2001141) | 0.52 | 1.74E-05 |

**Table S2. GO analysis of down-regulated genes in four galls**

| GO biological process | Fold Enrichment | P-value |
| --- | --- | --- |
| protein autophosphorylation (GO:0046777) | 8.98 | 1.78E-05 |
| protein phosphorylation (GO:0006468) | 5.56 | 3.16E-19 |
| phosphorylation (GO:0016310) | 5.39 | 1.16E-18 |
| response to salicylic acid (GO:0009751) | 4.84 | 5.00E-04 |
| signal transduction (GO:0007165) | 4.65 | 1.00E-31 |
| signaling (GO:0023052) | 4.5 | 9.52E-31 |
| cell communication (GO:0007154) | 4.1 | 7.91E-28 |
| defense response to bacterium (GO:0042742) | 3.88 | 1.85E-08 |
| regulation of defense response (GO:0031347) | 3.77 | 9.68E-06 |
| defense response (GO:0006952) | 3.68 | 2.55E-22 |
| defense response to fungus (GO:0050832) | 3.59 | 2.68E-05 |
| response to bacterium (GO:0009617) | 3.57 | 3.63E-09 |
| regulation of response to stress (GO:0080134) | 3.39 | 4.77E-05 |
| phosphate-containing compound metabolic process (GO:0006796) | 3.33 | 1.16E-10 |
| phosphorus metabolic process (GO:0006793) | 3.24 | 3.10E-10 |
| response to organic cyclic compound (GO:0014070) | 3.21 | 7.97E-04 |
| defense response to other organism (GO:0098542) | 3.11 | 7.49E-12 |
| cellular response to stimulus (GO:0051716) | 3.02 | 4.36E-22 |
| response to fungus (GO:0009620) | 2.91 | 1.10E-03 |
| response to external biotic stimulus (GO:0043207) | 2.84 | 3.85E-11 |
| response to other organism (GO:0051707) | 2.84 | 3.85E-11 |
| response to biotic stimulus (GO:0009607) | 2.84 | 4.05E-11 |
| biological process involved in interspecies interaction between organisms (GO:0044419) | 2.83 | 4.88E-11 |
| regulation of response to stimulus (GO:0048583) | 2.58 | 3.56E-03 |
| response to external stimulus (GO:0009605) | 2.45 | 1.16E-08 |
| protein modification process (GO:0036211) | 2.39 | 1.01E-07 |
| regulation of cellular process (GO:0050794) | 2.12 | 2.14E-13 |
| response to stress (GO:0006950) | 2.07 | 9.75E-11 |
| macromolecule modification (GO:0043412) | 2.01 | 6.68E-05 |
| response to stimulus (GO:0050896) | 1.89 | 1.28E-18 |
| regulation of biological process (GO:0050789) | 1.82 | 5.10E-11 |
| protein metabolic process (GO:0019538) | 1.8 | 1.72E-03 |
| biological regulation (GO:0065007) | 1.76 | 3.96E-10 |
| cellular process (GO:0009987) | 1.36 | 2.55E-06 |
| Unclassified (UNCLASSIFIED) | 0.94 | 0.00E+00 |

**Table S3. GO analysis up-regulated in hairy galls (Ketama and Wata)**

| GO biological process complete | Fold Enrichment | P-value |
| --- | --- | --- |
| cutin biosynthetic process (GO:0010143) | 8.96 | 2.17E-02 |
| cutin-based cuticle development (GO:0160062) | 7.53 | 3.89E-04 |
| DNA replication (GO:0006260) | 4.24 | 6.77E-04 |
| DNA-templated DNA replication (GO:0006261) | 4.17 | 8.40E-03 |
| mitotic cell cycle process (GO:1903047) | 3.66 | 2.47E-04 |
| chromosome organization (GO:0051276) | 3.59 | 1.93E-04 |
| mitotic cell cycle (GO:0000278) | 3.13 | 1.44E-03 |
| DNA repair (GO:0006281) | 2.95 | 9.54E-05 |
| DNA damage response (GO:0006974) | 2.78 | 1.72E-04 |
| chromatin remodeling (GO:0006338) | 2.74 | 1.68E-02 |
| cell cycle process (GO:0022402) | 2.73 | 3.14E-05 |
| cell cycle (GO:0007049) | 2.72 | 6.57E-06 |
| chromatin organization (GO:0006325) | 2.58 | 4.56E-02 |
| DNA metabolic process (GO:0006259) | 2.41 | 1.01E-03 |
| cellular response to stress (GO:0033554) | 1.72 | 3.48E-02 |
| cellular component organization (GO:0016043) | 1.59 | 2.26E-04 |
| nucleic acid metabolic process (GO:0090304) | 1.57 | 1.50E-02 |
| cellular component organization or biogenesis (GO:0071840) | 1.56 | 7.97E-05 |
| cellular aromatic compound metabolic process (GO:0006725) | 1.47 | 8.24E-03 |
| organic cyclic compound metabolic process (GO:1901360) | 1.45 | 1.26E-02 |
| cellular nitrogen compound metabolic process (GO:0034641) | 1.45 | 7.50E-03 |
| anatomical structure development (GO:0048856) | 1.43 | 1.93E-02 |
| developmental process (GO:0032502) | 1.4 | 4.34E-02 |
| organic substance biosynthetic process (GO:1901576) | 1.38 | 2.22E-02 |
| response to stimulus (GO:0050896) | 1.32 | 1.87E-03 |
| cellular process (GO:0009987) | 1.28 | 2.00E-09 |
| organic substance metabolic process (GO:0071704) | 1.26 | 1.82E-03 |
| primary metabolic process (GO:0044238) | 1.26 | 1.09E-02 |
| Unclassified (UNCLASSIFIED) | 0.88 | 0.00E+00 |

**Table S4. GO analysis down-regulated in hairy galls (Ketama and Wata)**

| GO biological process complete | Fold Enrichment | P-value |
| --- | --- | --- |
| photosynthesis, light reaction (GO:0019684) | 3.46 | 3.51E-03 |
| cellular response to hypoxia (GO:0071456) | 3.38 | 7.85E-07 |
| cellular response to decreased oxygen levels (GO:0036294) | 3.36 | 9.90E-07 |
| cellular response to oxygen levels (GO:0071453) | 3.34 | 1.11E-06 |
| photosynthesis (GO:0015979) | 3.17 | 3.33E-04 |
| response to hypoxia (GO:0001666) | 3.04 | 1.44E-05 |
| response to decreased oxygen levels (GO:0036293) | 2.99 | 2.14E-05 |
| response to oxygen levels (GO:0070482) | 2.97 | 2.60E-05 |
| response to heat (GO:0009408) | 2.83 | 5.99E-05 |
| response to jasmonic acid (GO:0009753) | 2.77 | 2.83E-02 |
| defense response to fungus (GO:0050832) | 2.72 | 5.64E-04 |
| response to fatty acid (GO:0070542) | 2.71 | 3.99E-02 |
| response to fungus (GO:0009620) | 2.64 | 1.47E-05 |
| response to wounding (GO:0009611) | 2.58 | 6.32E-03 |
| response to temperature stimulus (GO:0009266) | 2.36 | 4.03E-08 |
| regulation of defense response (GO:0031347) | 2.34 | 4.83E-02 |
| defense response to other organism (GO:0098542) | 2.19 | 5.66E-08 |
| response to biotic stimulus (GO:0009607) | 2.16 | 2.84E-11 |
| response to cold (GO:0009409) | 2.15 | 1.73E-02 |
| response to external biotic stimulus (GO:0043207) | 2.15 | 4.87E-11 |
| response to other organism (GO:0051707) | 2.15 | 4.87E-11 |
| biological process involved in interspecies interaction between organisms (GO:0044419) | 2.12 | 1.25E-10 |
| response to acid chemical (GO:0001101) | 2.07 | 2.63E-02 |
| defense response (GO:0006952) | 1.97 | 1.04E-06 |
| cellular response to stress (GO:0033554) | 1.96 | 2.42E-07 |
| response to osmotic stress (GO:0006970) | 1.93 | 3.36E-02 |
| response to external stimulus (GO:0009605) | 1.83 | 2.83E-08 |
| response to stress (GO:0006950) | 1.82 | 1.87E-20 |
| cellular response to chemical stimulus (GO:0070887) | 1.82 | 9.62E-05 |
| response to abiotic stimulus (GO:0009628) | 1.81 | 1.79E-11 |
| response to oxygen-containing compound (GO:1901700) | 1.69 | 8.10E-05 |
| cellular response to stimulus (GO:0051716) | 1.64 | 1.29E-08 |
| signal transduction (GO:0007165) | 1.61 | 6.30E-03 |
| signaling (GO:0023052) | 1.56 | 2.96E-02 |
| response to organic substance (GO:0010033) | 1.56 | 3.14E-03 |
| response to stimulus (GO:0050896) | 1.54 | 8.65E-18 |
| response to chemical (GO:0042221) | 1.49 | 1.15E-04 |
| regulation of biological process (GO:0050789) | 1.27 | 1.75E-02 |
| biological regulation (GO:0065007) | 1.26 | 8.22E-03 |
| cellular process (GO:0009987) | 1.25 | 6.02E-10 |
| metabolic process (GO:0008152) | 1.24 | 4.41E-05 |
| cellular metabolic process (GO:0044237) | 1.24 | 1.09E-02 |
| organic substance metabolic process (GO:0071704) | 1.24 | 6.21E-04 |
| primary metabolic process (GO:0044238) | 1.23 | 4.95E-03 |
| Unclassified (UNCLASSIFIED) | 0.98 | 0.00E+00 |

**Table S5. GO analysis of up-regulated genes in five galls**

| GO biological process complete | Fold enrichment | P-value |
| --- | --- | --- |
| cellular response to gravity (GO:0071258) | 38.43 | 4.90E-02 |
| gluconeogenesis (GO:0006094) | 15.04 | 8.58E-06 |
| maturation of LSU-rRNA from tricistronic rRNA transcript (GO:0000463) | 14.16 | 8.42E-04 |
| cytoplasmic translation (GO:0002181) | 13.47 | 3.64E-20 |
| hexose biosynthetic process (GO:0019319) | 13.3 | 3.06E-05 |
| ATP biosynthetic process (GO:0006754) | 11.3 | 2.79E-05 |
| proton motive force-driven ATP synthesis (GO:0015986) | 11.3 | 2.79E-05 |
| purine ribonucleoside triphosphate biosynthetic process (GO:0009206) | 10.98 | 1.33E-06 |
| translational elongation (GO:0006414) | 10.84 | 8.29E-06 |
| purine nucleoside triphosphate biosynthetic process (GO:0009145) | 10.72 | 1.80E-06 |
| glucose metabolic process (GO:0006006) | 10.41 | 5.25E-07 |
| ribonucleoside triphosphate biosynthetic process (GO:0009201) | 9.96 | 2.00E-07 |
| protein refolding (GO:0042026) | 9.61 | 7.30E-04 |
| nucleoside triphosphate biosynthetic process (GO:0009142) | 9.44 | 4.40E-07 |
| maturation of LSU-rRNA (GO:0000470) | 9.37 | 2.02E-04 |
| purine ribonucleoside triphosphate metabolic process (GO:0009205) | 9.32 | 1.72E-13 |
| ATP metabolic process (GO:0046034) | 9.29 | 3.64E-12 |
| ribonucleoside triphosphate metabolic process (GO:0009199) | 9 | 2.18E-14 |
| purine nucleoside triphosphate metabolic process (GO:0009144) | 8.95 | 4.60E-13 |
| oxidative phosphorylation (GO:0006119) | 8.87 | 2.55E-07 |
| long-chain fatty acid biosynthetic process (GO:0042759) | 8.78 | 7.14E-03 |
| ribosomal large subunit biogenesis (GO:0042273) | 8.64 | 3.22E-10 |
| ribonucleoside monophosphate biosynthetic process (GO:0009156) | 8.43 | 2.42E-03 |
| nucleoside triphosphate metabolic process (GO:0009141) | 8.4 | 1.38E-13 |
| proteinogenic amino acid catabolic process (GO:0170040) | 8.35 | 6.54E-04 |
| L-amino acid catabolic process (GO:0170035) | 8.35 | 6.54E-04 |
| ribonucleoside monophosphate metabolic process (GO:0009161) | 8.23 | 3.01E-03 |
| monosaccharide biosynthetic process (GO:0046364) | 8.18 | 8.11E-04 |
| ADP catabolic process (GO:0046032) | 8.13 | 2.20E-04 |
| ribonucleoside diphosphate catabolic process (GO:0009191) | 8.13 | 2.20E-04 |
| purine ribonucleoside diphosphate catabolic process (GO:0009181) | 8.13 | 2.20E-04 |
| purine nucleoside diphosphate catabolic process (GO:0009137) | 8.13 | 2.20E-04 |
| glycolytic process (GO:0006096) | 8.13 | 2.20E-04 |
| fatty acid beta-oxidation (GO:0006635) | 8.09 | 1.39E-02 |
| chaperone cofactor-dependent protein refolding (GO:0051085) | 8.01 | 1.00E-03 |
| 'de novo' post-translational protein folding (GO:0051084) | 8.01 | 1.00E-03 |
| protein folding (GO:0006457) | 7.97 | 1.46E-20 |
| ADP metabolic process (GO:0046031) | 7.83 | 3.32E-04 |
| purine ribonucleoside diphosphate metabolic process (GO:0009179) | 7.83 | 3.32E-04 |
| purine nucleoside diphosphate metabolic process (GO:0009135) | 7.83 | 3.32E-04 |
| pyridine nucleotide catabolic process (GO:0019364) | 7.83 | 3.32E-04 |
| ribonucleoside diphosphate metabolic process (GO:0009185) | 7.82 | 8.99E-05 |
| purine ribonucleotide biosynthetic process (GO:0009152) | 7.76 | 2.03E-10 |
| ATP synthesis coupled electron transport (GO:0042773) | 7.69 | 4.05E-04 |
| ribonucleotide catabolic process (GO:0009261) | 7.55 | 4.92E-04 |
| purine ribonucleotide catabolic process (GO:0009154) | 7.55 | 4.92E-04 |
| pyridine-containing compound catabolic process (GO:0072526) | 7.55 | 4.92E-04 |
| mitochondrial ATP synthesis coupled electron transport (GO:0042775) | 7.52 | 6.77E-03 |
| nucleoside monophosphate biosynthetic process (GO:0009124) | 7.52 | 6.77E-03 |
| aerobic electron transport chain (GO:0019646) | 7.52 | 6.77E-03 |
| ribonucleotide biosynthetic process (GO:0009260) | 7.51 | 2.47E-12 |
| chaperone-mediated protein folding (GO:0061077) | 7.46 | 4.39E-05 |
| respiratory electron transport chain (GO:0022904) | 7.44 | 1.62E-04 |
| lipid oxidation (GO:0034440) | 7.39 | 2.20E-03 |
| pyruvate metabolic process (GO:0006090) | 7.37 | 1.44E-05 |
| nucleoside monophosphate metabolic process (GO:0009123) | 7.36 | 8.18E-03 |
| fatty acid oxidation (GO:0019395) | 7.32 | 3.05E-02 |
| hexose metabolic process (GO:0019318) | 7.26 | 4.24E-07 |
| 'de novo' protein folding (GO:0006458) | 7.25 | 2.65E-03 |
| ribose phosphate biosynthetic process (GO:0046390) | 7.24 | 6.31E-12 |
| nucleoside diphosphate catabolic process (GO:0009134) | 7.16 | 8.62E-04 |
| purine nucleotide biosynthetic process (GO:0006164) | 7.15 | 1.01E-10 |
| response to zinc ion (GO:0010043) | 7.06 | 1.18E-02 |
| fatty acid catabolic process (GO:0009062) | 6.99 | 4.38E-02 |
| ribosome assembly (GO:0042255) | 6.99 | 3.35E-04 |
| purine-containing compound biosynthetic process (GO:0072522) | 6.93 | 2.05E-10 |
| purine nucleotide catabolic process (GO:0006195) | 6.93 | 1.23E-03 |
| nucleoside diphosphate metabolic process (GO:0009132) | 6.88 | 3.98E-04 |
| translation (GO:0006412) | 6.76 | 2.91E-47 |
| peptide biosynthetic process (GO:0043043) | 6.69 | 8.02E-47 |
| purine ribonucleotide metabolic process (GO:0009150) | 6.66 | 1.48E-15 |
| alpha-amino acid catabolic process (GO:1901606) | 6.64 | 5.87E-05 |
| nucleoside phosphate biosynthetic process (GO:1901293) | 6.63 | 1.75E-12 |
| nucleotide biosynthetic process (GO:0009165) | 6.63 | 1.75E-12 |
| nucleotide catabolic process (GO:0009166) | 6.6 | 2.04E-03 |
| ribonucleotide metabolic process (GO:0009259) | 6.6 | 1.90E-17 |
| translational initiation (GO:0006413) | 6.59 | 6.58E-04 |
| amino acid catabolic process (GO:0009063) | 6.48 | 2.63E-05 |
| aerobic respiration (GO:0009060) | 6.45 | 1.02E-08 |
| amide biosynthetic process (GO:0043604) | 6.41 | 8.20E-49 |
| long-chain fatty acid metabolic process (GO:0001676) | 6.4 | 8.71E-03 |
| ribose phosphate metabolic process (GO:0019693) | 6.17 | 2.34E-16 |
| purine-containing compound catabolic process (GO:0072523) | 6.15 | 1.43E-03 |
| peptide metabolic process (GO:0006518) | 6.11 | 2.82E-46 |
| energy derivation by oxidation of organic compounds (GO:0015980) | 6.1 | 1.48E-10 |
| monocarboxylic acid catabolic process (GO:0072329) | 6 | 1.59E-02 |
| cellular respiration (GO:0045333) | 5.95 | 6.59E-09 |
| nucleoside phosphate catabolic process (GO:1901292) | 5.87 | 6.85E-03 |
| protein maturation (GO:0051604) | 5.78 | 3.94E-17 |
| amide metabolic process (GO:0043603) | 5.77 | 3.65E-49 |
| purine nucleotide metabolic process (GO:0006163) | 5.75 | 3.74E-16 |
| organic acid catabolic process (GO:0016054) | 5.56 | 2.97E-08 |
| carboxylic acid catabolic process (GO:0046395) | 5.56 | 2.97E-08 |
| nucleotide metabolic process (GO:0009117) | 5.47 | 1.70E-17 |
| purine-containing compound metabolic process (GO:0072521) | 5.47 | 8.97E-16 |
| ribosomal small subunit biogenesis (GO:0042274) | 5.37 | 2.41E-03 |
| monosaccharide metabolic process (GO:0005996) | 5.36 | 2.02E-05 |
| nicotinamide nucleotide metabolic process (GO:0046496) | 5.3 | 1.62E-04 |
| nucleoside phosphate metabolic process (GO:0006753) | 5.29 | 6.84E-17 |
| cellular lipid catabolic process (GO:0044242) | 5.29 | 4.33E-04 |
| pyridine nucleotide metabolic process (GO:0019362) | 5.25 | 1.84E-04 |
| lipid catabolic process (GO:0016042) | 5.12 | 2.63E-04 |
| small molecule catabolic process (GO:0044282) | 4.84 | 7.17E-09 |
| pyridine-containing compound metabolic process (GO:0072524) | 4.84 | 5.86E-04 |
| response to endoplasmic reticulum stress (GO:0034976) | 4.8 | 8.75E-03 |
| ribosome biogenesis (GO:0042254) | 4.79 | 2.26E-14 |
| nucleobase-containing small molecule metabolic process (GO:0055086) | 4.75 | 1.32E-17 |
| organophosphate catabolic process (GO:0046434) | 4.71 | 2.59E-02 |
| protein-RNA complex assembly (GO:0022618) | 4.42 | 2.03E-03 |
| cellular nitrogen compound biosynthetic process (GO:0044271) | 4.37 | 7.09E-50 |
| ribonucleoprotein complex biogenesis (GO:0022613) | 4.28 | 7.10E-14 |
| protein-RNA complex organization (GO:0071826) | 4.27 | 3.28E-03 |
| generation of precursor metabolites and energy (GO:0006091) | 4.23 | 5.33E-12 |
| fatty acid biosynthetic process (GO:0006633) | 4.1 | 5.65E-03 |
| response to heat (GO:0009408) | 4.07 | 1.27E-07 |
| monocarboxylic acid metabolic process (GO:0032787) | 4.06 | 2.43E-12 |
| organonitrogen compound biosynthetic process (GO:1901566) | 3.96 | 6.86E-44 |
| fatty acid metabolic process (GO:0006631) | 3.87 | 1.31E-05 |
| rRNA processing (GO:0006364) | 3.69 | 1.30E-04 |
| rRNA metabolic process (GO:0016072) | 3.65 | 4.59E-05 |
| organophosphate biosynthetic process (GO:0090407) | 3.46 | 3.72E-06 |
| gene expression (GO:0010467) | 3.46 | 2.67E-45 |
| carboxylic acid metabolic process (GO:0019752) | 3.2 | 1.56E-14 |
| nucleobase-containing compound biosynthetic process (GO:0034654) | 3.17 | 9.45E-08 |
| carbohydrate derivative biosynthetic process (GO:1901137) | 3.15 | 2.85E-06 |
| cellular catabolic process (GO:0044248) | 3.13 | 7.33E-10 |
| response to metal ion (GO:0010038) | 3.11 | 2.33E-02 |
| organophosphate metabolic process (GO:0019637) | 3.08 | 1.78E-08 |
| aromatic compound catabolic process (GO:0019439) | 3.07 | 1.74E-02 |
| ncRNA metabolic process (GO:0034660) | 3.04 | 1.03E-06 |
| small molecule metabolic process (GO:0044281) | 3.02 | 1.14E-23 |
| oxoacid metabolic process (GO:0043436) | 2.98 | 1.52E-13 |
| organic acid metabolic process (GO:0006082) | 2.98 | 1.61E-13 |
| macromolecule biosynthetic process (GO:0009059) | 2.97 | 1.24E-39 |
| organonitrogen compound catabolic process (GO:1901565) | 2.96 | 2.68E-12 |
| proteasomal protein catabolic process (GO:0010498) | 2.96 | 1.91E-02 |
| organic cyclic compound catabolic process (GO:1901361) | 2.96 | 3.08E-02 |
| response to temperature stimulus (GO:0009266) | 2.91 | 1.37E-08 |
| carbohydrate derivative metabolic process (GO:1901135) | 2.88 | 4.91E-09 |
| ncRNA processing (GO:0034470) | 2.87 | 5.11E-04 |
| amino acid metabolic process (GO:0006520) | 2.76 | 2.86E-03 |
| organic substance biosynthetic process (GO:1901576) | 2.72 | 1.42E-54 |
| cellular biosynthetic process (GO:0044249) | 2.7 | 3.20E-51 |
| biosynthetic process (GO:0009058) | 2.64 | 2.04E-52 |
| organonitrogen compound metabolic process (GO:1901564) | 2.64 | 1.11E-57 |
| response to salt stress (GO:0009651) | 2.61 | 1.82E-03 |
| heterocycle biosynthetic process (GO:0018130) | 2.6 | 2.55E-06 |
| response to osmotic stress (GO:0006970) | 2.57 | 2.61E-04 |
| organic substance catabolic process (GO:1901575) | 2.57 | 1.03E-12 |
| protein metabolic process (GO:0019538) | 2.55 | 7.52E-35 |
| cellular nitrogen compound metabolic process (GO:0034641) | 2.53 | 3.45E-33 |
| catabolic process (GO:0009056) | 2.49 | 2.32E-13 |
| proteolysis involved in protein catabolic process (GO:0051603) | 2.48 | 1.37E-03 |
| response to inorganic substance (GO:0010035) | 2.48 | 5.25E-06 |
| protein-containing complex assembly (GO:0065003) | 2.46 | 9.58E-03 |
| small molecule biosynthetic process (GO:0044283) | 2.41 | 1.49E-03 |
| modification-dependent macromolecule catabolic process (GO:0043632) | 2.41 | 2.60E-02 |
| protein catabolic process (GO:0030163) | 2.39 | 2.48E-03 |
| modification-dependent protein catabolic process (GO:0019941) | 2.39 | 4.12E-02 |
| cellular component biogenesis (GO:0044085) | 2.36 | 2.59E-09 |
| aromatic compound biosynthetic process (GO:0019438) | 2.34 | 1.37E-04 |
| organic cyclic compound biosynthetic process (GO:1901362) | 2.33 | 2.09E-05 |
| proteolysis (GO:0006508) | 2.29 | 8.82E-04 |
| nitrogen compound metabolic process (GO:0006807) | 2.16 | 7.68E-49 |
| cellular metabolic process (GO:0044237) | 2.15 | 2.29E-53 |
| primary metabolic process (GO:0044238) | 2.09 | 7.65E-56 |
| protein-containing complex organization (GO:0043933) | 2.07 | 1.89E-02 |
| organic substance metabolic process (GO:0071704) | 2.03 | 1.28E-57 |
| metabolic process (GO:0008152) | 1.96 | 6.69E-58 |
| lipid metabolic process (GO:0006629) | 1.96 | 3.51E-02 |
| macromolecule metabolic process (GO:0043170) | 1.93 | 1.99E-26 |
| response to abiotic stimulus (GO:0009628) | 1.93 | 4.66E-08 |
| nucleobase-containing compound metabolic process (GO:0006139) | 1.87 | 2.37E-07 |
| cellular response to stress (GO:0033554) | 1.83 | 4.22E-02 |
| reproductive structure development (GO:0048608) | 1.8 | 3.50E-02 |
| reproductive system development (GO:0061458) | 1.8 | 3.57E-02 |
| cellular aromatic compound metabolic process (GO:0006725) | 1.8 | 9.25E-08 |
| organic cyclic compound metabolic process (GO:1901360) | 1.79 | 5.59E-08 |
| heterocycle metabolic process (GO:0046483) | 1.78 | 8.90E-07 |
| cellular component organization or biogenesis (GO:0071840) | 1.77 | 2.63E-07 |
| post-embryonic development (GO:0009791) | 1.76 | 1.11E-02 |
| response to stress (GO:0006950) | 1.75 | 3.41E-09 |
| phosphorus metabolic process (GO:0006793) | 1.72 | 3.90E-02 |
| cellular process (GO:0009987) | 1.69 | 9.23E-51 |
| response to chemical (GO:0042221) | 1.68 | 2.94E-05 |
| system development (GO:0048731) | 1.66 | 8.44E-03 |
| multicellular organism development (GO:0007275) | 1.58 | 4.76E-03 |
| response to stimulus (GO:0050896) | 1.53 | 2.88E-09 |
| anatomical structure development (GO:0048856) | 1.5 | 1.30E-02 |
| multicellular organismal process (GO:0032501) | 1.5 | 2.67E-02 |
| developmental process (GO:0032502) | 1.47 | 4.08E-02 |
| biological_process (GO:0008150) | 1.11 | 4.18E-07 |
| Unclassified (UNCLASSIFIED) | 0.56 | 0.00E+00 |
| regulation of nucleobase-containing compound metabolic process (GO:0019219) | 0.46 | 5.88E-03 |
| regulation of RNA metabolic process (GO:0051252) | 0.37 | 4.73E-05 |
| regulation of DNA-templated transcription (GO:0006355) | 0.29 | 1.25E-06 |
| regulation of RNA biosynthetic process (GO:2001141) | 0.29 | 1.26E-06 |

**Table S6. GO analysis of down-regulated genes in five galls**

| GO biological process complete | Fold enrichment | P-value |
| --- | --- | --- |
| protein autophosphorylation (GO:0046777) | 13.05 | 1.39E-04 |
| protein phosphorylation (GO:0006468) | 7.67 | 3.43E-07 |
| defense response (GO:0006952) | 7.29 | 4.66E-16 |
| defense response to bacterium (GO:0042742) | 6.89 | 2.19E-03 |
| phosphorylation (GO:0016310) | 6.76 | 6.02E-08 |
| response to bacterium (GO:0009617) | 6.23 | 6.29E-04 |
| signal transduction (GO:0007165) | 5.93 | 1.63E-14 |
| signaling (GO:0023052) | 5.75 | 4.05E-14 |
| defense response to other organism (GO:0098542) | 5.55 | 1.76E-06 |
| cell communication (GO:0007154) | 4.98 | 3.11E-12 |
| response to external biotic stimulus (GO:0043207) | 4.25 | 6.34E-05 |
| response to other organism (GO:0051707) | 4.25 | 6.34E-05 |
| response to biotic stimulus (GO:0009607) | 4.24 | 6.61E-05 |
| biological process involved in interspecies interaction between organisms (GO:0044419) | 4.2 | 7.69E-05 |
| phosphate-containing compound metabolic process (GO:0006796) | 3.49 | 1.64E-03 |
| phosphorus metabolic process (GO:0006793) | 3.38 | 2.65E-03 |
| cellular response to stimulus (GO:0051716) | 3.36 | 5.42E-08 |
| response to external stimulus (GO:0009605) | 3.2 | 3.53E-03 |
| protein modification process (GO:0036211) | 2.99 | 5.95E-03 |
| response to stress (GO:0006950) | 2.83 | 1.15E-06 |
| macromolecule modification (GO:0043412) | 2.57 | 4.35E-02 |
| response to stimulus (GO:0050896) | 2.5 | 3.09E-10 |
| regulation of cellular process (GO:0050794) | 2.22 | 2.04E-04 |
| regulation of biological process (GO:0050789) | 2.09 | 6.90E-04 |
| biological regulation (GO:0065007) | 1.97 | 3.18E-03 |
| cellular process (GO:0009987) | 1.59 | 9.93E-04 |
| Unclassified (UNCLASSIFIED) | 0.47 | 0.00E+00 |

**Table S7. Sample preparation and scanning protocol for each specimen**

|  | **Staining** | | **MicroCT Scanning** | | | | | | | | | | **Data analysis** | | |
| --- | --- | --- | --- | --- | --- | --- | --- | --- | --- | --- | --- | --- | --- | --- | --- |
| **Strain** | **solution** | **Time** | **Scanning device** | **Scanning medium** | **Scanned parts** | **Scanned method** | **Voltage and current** | **Frame average** | **Frame rate** | **Rotation steps** | **Number of projections** | **Voxel size ( µm )*** | **Dataset resolution ( µm )*** | **Dataset size ( GB )**** | **Figure and Movie** |
| **Eboshi** | 25% Lugol | 8 days | ScanXmate-E090S105 | air | whole | Normal | 85kV 90µA | 2 | 8 fps | 0.3゜ | 1200 | 10.0 | 10.0 | 0.52 | Fig2B , Movie1 |
|  |  |  |  | air | insect | Normal | 85kV 90µA | 4 | 8 fps | 0.24゜ | 1500 | 2.8 | 2.8 | 0.96 | Fig3D, Movie11 |
|  | 25% Lugol 　 (re-staining) | 1hr | ScanXmate-CF110TSH320/460 | 0.5% agarose | whole | Multi-step | 50kV 70µA | 4 | 12 fps | 0.18゜ | 2000 | 2.8 | 2.8 | 4.99 | Fig3A, Movie10 |
|  | 25% Lugol | 49 days | ScanXmate-E090S105 | 0.5% agarose | whole | Normal | 85kV 90µA | 2 | 8 fps | 0.24゜ | 1500 | 6.4 | 6.4 | 0.90 | Fig3F, Movie12 |
|  | 25% Lugol 　 (re-staining) | 5 days | ScanXmate-CF110TSH320/460 | 0.5% agarose | whole | Multi-step | 50kV 70µA | 4 | 12 fps | 0.18゜ | 2000 | 2.8 | 2.8 | 4.47 | Fig3B, Movie10 |
|  | 25% Lugol | 49 days | ScanXmate-E090S105 | 0.5% agarose | whole | Multi-step | 85kV 90µA | 2 | 8 fps | 0.24゜ | 1500 | 4.8 | 4.8 | 1.34 | Fig2A , Fig3G |
|  | 25% Lugol 　 (re-staining) | 5 days | ScanXmate-CF110TSH320/460 | 0.5% agarose | whole | Multi-step | 50kV 70µA | 4 | 12 fps | 0.18゜ | 2000 | 2.8 | 2.8 | 4.77 | Fig3C, Movie10 |
|  | 3% PTA | 2 days | ScanXmate-CF110TSH320/460 | 0.5% agarose | whole | Multi-step | 50kV 70µA | 4 | 12 fps | 0.18゜ | 2000 | 2.3 | 3.8 | 1.57 | Fig2C, Movie2 |
|  | 25% Lugol | 7 days | ScanXmate-CF110TSH320/460 | 0.5% agarose | whole | Multi-step | 50kV 70µA | 4 | 12 fps | 0.18゜ | 2000 | 2.5 | 4.2 | 1.00 | Fig2D, Movie3 |
| **Ketama** | 25% Lugol | 38 days | ScanXmate-E090S105 | air | whole | Multi-step | 85kV 90µA | 2 | 8 fps | 0.24゜ | 1500 | 9.0 | 9.0 | 1.29 | Fig2E |
|  |  |  |  |  |  |  |  |  |  |  |  |  | 15.0 | 0.28 | Fig2F,G, Movie4 |
|  |  |  |  | 0.5% agarose | base of galls | Normal | 85kV 90µA | 4 | 8 fps | 0.18゜ | 2000 | 5.5 | 5.5 | 0.96 | Fig3H, Movie13 |
|  | 25% Lugol | 38 days | ScanXmate-E090S105 | air | whole | Normal | 85kV 90µA | 2 | 8 fps | 0.24゜ | 1500 | 5.3 | 5.3 | 0.91 | Fig2H |
| **Cobu** | 25% Lugol | 49 days | ScanXmate-E090S105 | 0.5% agarose | whole | Multi-step | 85kV 90µA | 2 | 8 fps | 0.24゜ | 1500 | 10.0 | 10.0 | 1.24 | Fig2I ,J, Movie5 |
|  |  |  |  |  |  |  |  |  |  |  |  |  | 16.7 | 0.27 | Fig2K, L, Movie6 |
|  |  |  |  | 0.5% agarose | insect | Normal | 85kV 90µA | 4 | 8 fps | 0.2゜ | 1800 | 3.9 | 3.9 | 0.94 | Fig3I, Movie14 |
| **Wata** | 25% Lugol | 49 days | ScanXmate-E090S105 | 0.5% agarose | whole | Multi-step | 85kV 90µA | 2 | 8 fps | 0.24゜ | 1500 | 9.0 | 9.0 | 1.16 | Fig2M,N , Movie7 |
|  |  |  |  |  |  |  |  |  |  |  |  |  | 15.0 | 0.25 | Fig2O, Fig3K, Movie8 |
|  |  |  |  | 0.5% agarose | insect | Normal | 85kV 90µA | 4 | 8 fps | 0.2゜ | 1800 | 3.8 | 3.8 | 0.93 | Fig3J |
|  | 25% Lugol (re-staining) | 5 days | ScanXmate-CF110TSH320/460 | 0.5% agarose | whole | Multi-step | 50kV 70µA | 4 | 12 fps | 0.18゜ | 2000 | 2.8 | 4.7 | 1.48 | Fig2P, Movie9 |
| **Metsubo** | 25% Lugol | 38 days | ScanXmate-E090S105 | 0.5% agarose | whole | Multi-step | 85kV 90µA | 2 | 8 fps | 0.24゜ | 1500 | 12.5 | 20.8 | 0.27 | Fig5G , H |
|  | 25% Lugol | 38 days | ScanXmate-E090S105 | 0.5% agarose | insect | Normal | 85kV 90µA | 4 | 8 fps | 0.18゜ | 2000 | 3.8 | 3.8 | 0.96 | Fig5I , J |
